# The polarity protein Dlg5 regulates collective cell migration during *Drosophila* oogenesis

**DOI:** 10.1371/journal.pone.0226061

**Published:** 2019-12-19

**Authors:** Jun Luo, Ping Zhou, Xuan Guo, Dou Wang, Jiong Chen

**Affiliations:** 1 College of Life Science, Shangrao Normal University, Shangrao, China; 2 State Key Laboratory of Pharmaceutical Biotechnology and MOE Key Laboratory of Model Animals for Disease Study, Model Animal Research Center, Nanjing University, Nanjing, China; 3 State Key Laboratory of Molecular Developmental Biology, Institute of Genetics and Developmental Biology, Chinese Academy of Sciences, Beijing, China; University of Dayton, UNITED STATES

## Abstract

Collective migration plays critical roles in animal development, physiological events, and cancer metastasis. However, the molecular mechanisms of collective cell migration are not well understood. *Drosophila* border cells represent an excellent *in vivo* genetic model to study collective cell migration and identify novel regulatory genes for cell migration. Using the Mosaic Analysis with a Repressible Cell Marker (MARCM) system, we screened 240 P-element insertion lines to identify essential genes for border cell migration. Two genes were uncovered, including *dlg5* (*discs large 5*) and *CG31689*. Further analysis showed that Dlg5 regulates the apical-basal polarity and cluster integrity in border cell clusters. Dlg5 is enriched in lateral surfaces between border cells and central polar cells but also shows punctate localization between border cells. We found that the distribution of Dlg5 in border cell clusters is regulated by Armadillo. Structure-function analysis revealed that the N-terminal Coiled-coil domain and the C-terminal PDZ3-PDZ4-SH3-GUK domains but not the PDZ1-PDZ2 domains of Dlg5 are required for BC migration. The Coiled-coil domain and the PDZ4-SH3-GUK domains are critical for Dlg5’s cell surface localization in border cell clusters.

## Introduction

Cell migration plays a critical role in embryonic development, wound healing and physiological processes. Clarifying the molecular mechanism of cell migration helps better understand the abnormal cell migration involved pathological processes such as tumor metastasis and inflammation. Cells can move singly, they also can migrate as groups in the form of collective migration [1]. Collective migration has been observed in embryo gastrulation, wound healing and tumor metastasis [1–3]. During collective migration, how individual cells interact with each other and maintain coherence as a collective group during migration is poorly understood. The BC migration in *Drosophila* ovary is an excellent and genetically tractable system to study collective cell migration [2, 4, 5]. At early stage 9 of *Drosophila* oogenesis, border cells (BCs) are first specified and selected out from the anterior follicle epithelium that contain inherent apical-basal polarity [5]. The BC cluster contains two non-migratory polar cells (PCs) in the center and about six BCs surrounding the PCs (S1A Fig). Then, the BCs detach from anterior the follicle epithelium, invade into the nurse cells, migrate for about 150um and reach the border between oocyte and nurse cells by early stage 10 (S1A Fig). Previous works have identified many of the important genes required for BC migration, such as Slbo, Pvf1, Apontic, Abrupt, Par1, Taiman, Jing, Psidin, Stat92E, Puckered, and Sec3 [6–15]. The roles of several apical and basolateral polarity components have also been analyzed in BCs, including Par complex components Baz/Par3 and Par6, the adherens junctional molecule E-cadherin (Ecad), and the basolateral complex components Dlg and Lgl [16–20]. However, how the apical-basal polarity proteins are regulated in BC clusters are still not well understood.

Forward genetic screen in *Drosophila* is a powerful way to identify novel genes involved in biological processes. A large collection of P-element insertion stocks was available from the Berkeley *Drosophila* Genome Project [21]. Homozygous P-element mutants of most important genes are lethal and cannot be used to study biological processes in adult flies directly. The Flp/FRT (Flippase/Flp recombination target) system has been developed to generate mosaic flies for the lethal gene analysis [22, 23]. The MARCM (Mosaic Analysis with a Repressible Cell Marker) system is an improved Flp/FRT system, which combines the Gal4/UAS, Gal80 and Flp/FRT systems to positively label the mutant cell with GFP [24]. Using MARCM system, the mutant clones can be easily observed with the expressed GFP marker. Here, we utilized the UCLA “Bruinfly” FRT40A-lethal P collection and conducted a P-element based loss-of-function screen to uncover novel genes required for BC migration [25]. Overall, *dlg5* (*discs large 5*) and *CG31689* are two genes recovered from this screen.

Discs large 5 (Dlg5) is a member of membrane-associated guanylate kinase (MAGUK) super family which contains a core of three domains in conserved sequential order, PDZ-SH3-GUK [26]. Dlg5 is highly conserved in human, mouse, chicken, zebra fish and *Drosophila* [27]. The cell polarity proteins Dlg and Sdt are also MAGUK members, which function as molecular scaffolds in basolateral complex (Dlg/Lgl/Scrib) and apical Crumb complex (Crb/Sdt/PATJ) respectively. Human Dlg5 was expressed in prostate gland epithelia and in placenta [28]. Dlg5 in vertebrate interacted with a variety of proteins, such as β-catenin, vinexin, P55, Citron kinase, Girdin, Syntaxin, TGF-β receptors and Smoothened [27–33]. But most of these studies werebased on cell culture. The first genetic analysis of *Dlg5* was based on *Dlg5* knockout mice, which displayed failure of epithelial tube maintenance in brain and kidneys, and abnormal lung morphogenesis [27, 34]. RNAi knockdown of *dlg5* in *Drosophila* affected the migration and morphology of BC clusters [35]. Recently, it has been reported that *Drosophila dlg5* mutation caused loss of germ cells and embryonic lethality [36]. Moreover, RNAi knockdown of *dlg5* in the follicle cells causes defects in stalk cell overgrowth, egg chamber budding, and ectopic PC induction [36]. Our previous study in *Drosophila* has revealed that Dlg5 plays an essential role in apical polarity maintenance in follicular epithelia [37]. The previous work about Dlg5 in BC migration study is based on RNAi knockdown, the localization and function of Dlg5 in BC migration have not been extensively addressed. Here, we report that a genetic screen for BC migration has identified the *Drosophila* Dlg5 as an essential player for regulation of apical-basal polarity of BC clusters. The expression, distribution and structure-function of Dlg5 in BC clusters are reported in this study.

## Materials and methods

### *Drosophila* genetics

Flies were cultured following standard procedures at 25°C except for RNAi experiments at 29°C. All strains were obtained from the Bloomington *Drosophila* Stock Center, except for the following: *dlg5*^*EP2087*^*/CyO* (Szeged stock Center), *Ecad-GFP* [38], *UAS-Par6*.*mCherry* [39], *UAS-aPKC* [40]. *UAS-Dcr2* was kindly provided by Dr. Barry Dickson. The GFP trap lines, *Nrg-GFP*, *Jupiter-GFP*, *GalT-GFP*, *ATPalpha-GFP*, *Lac-GFP* and *CG31689*^*CB03239*^, were gifts from Dr. Lynn Cooley. *USG-Gal4* was kindly provided by Dr. Stephane Noselli [41]. All the RNAi lines were obtained from Vienna *Drosophila* RNAi Center. The *UAS-dlg5*.*RNAi* strains employed were: GD22496, GD46234 and KK101596 (only the first one was shown). The *UAS-aPKC*.*RNAi*, *UAS-Par6*.*RNAi* and *UAS-Arm*.*RNAi* lines were KK100874, KK101015 and KK102545 respectively. *Ubi-Dlg5*.*EGFP*, *Dlg5-TagRFP-T* and all the *dlg5* truncated transgenes (Δ1, Δ2, Δ3, Δ4, Δ5, Δ6, Δ7, Δ8, Δ9, C1, C2, C3, C4, N1, N2, N3, N4, M1, M2, M3, M4) were descripted previously [37]. The FRT P-element insertion lethal lines used in this study were obtained from the Howard Hughes Medical Institute. The *dlg5*^*KG748*^ mutant was backcrossed to isogenized *w*^*1118*^ for more than 15 generations to outcross the background mutations. Clonal analyses in the adult ovaries were performed using the hs-Flp/FRT systems with GFP or RFP as marker for wild-type cells. Three days after eclosion, adult flies were heat-shocked at 37°C for one hour every day in three days and then fed with fresh yeast for one day before dissection. Experiments with the temperature sensitive Gal80^ts^ system were carried out at 18°C to repress GAL4-mediated transcriptional activation. Three days after eclosion, adult flies were transferred to 29°C and induced for three days before dissection. Genotype list was provided in S1 Table.

### P-element Screen with MARCM system

The MARCM tool *yw*, *hs-Flp*, *tub-Gal4*, *UAS-mCD8*::*GFP; tub-Gal80*, *FRT40A/CyO* was used to generate GFP positive MARCM clones [24]. To perform the screen, the virgins of *yw*, *hs-Flp*, *tub-Gal4*, *UAS-mCD8*::*GFP; tub-Gal80*, *FRT40A/CyO* were collected and mated to FRT-P-element lethal lines. Then the 2^nd^ and 3^rd^ instar larvae from these crosses were heat-shocked at 37°C for two hours every day in three days. After eclosion, non-curly females were fed with fresh yeast for 2–3 days. Ovaries were dissected in PBS and mounted in medium. For BC migration examination, fluorescent images of stage 10 egg chambers was taken using Olympus BX51 fluorescent microscopy. According to the position of BC clusters at stage 10, each egg chamber was classified into two classes, Normal or Delay. The Normal class was identified when BC cluster reached the border between nurse cells and the oocyte at stage 10. The Delay class was identified when BC cluster did not reach the border and stayed among nurse cells at stage 10.

### Transgenes

To generate the ubiquitous expression construct *Ubi-Dlg5*.*TagRFP-T*, Dlg5 CDS was amplified from LD32687 (DGRC) and subcloned into the vector pUbRPT.attB [42]. After sequencing, the construct was inserted into the ZH-51C attP docking site using established PhiC31-based methods. The generation of the genomic fragment transgene *Dlg5-TagRFP-T*, the GFP tagged transgene *Ubi-Dlg5*.*EGFP* and all the *dlg5* truncated transgenes were descripted previously [37].

### Immunohistochemistry and microscopy

Ovary dissection was carried out in phosphate-buffered saline (PBS) and then fixed in Devitellinizing buffer (7% formaldehyde) and heptane (Sigma) mixture (1:6) for 10min. After washes in PBS, ovaries were incubated in blocking solution (PBT, 10% goat serum) for 30min and then stained overnight at 4°C. Primary antibodies and their concentrations were as follows: mouse anti-Arm (1:50; N27A1; Developmental Studies Hybridoma Bank (DSHB)), rabbit anti-PKCζ (1:200; C-20; Santa Cruz), mouse anti-Dlg (1:50; 4F3; DSHB), mouse anti-Crb (1:10; Cq4; DSHB), rat anti-Ncad (1:20; DN-Ex #8; DSHB). Methanol treatment was used after fixation for anti-Crb staining. After washes in PBT, ovaries were incubated with secondary antibodies (1:250, Jackson ImmunoResearch) for 2 hours at room temperature. F-actin was labeled by Rhodamine phalloidin (1:150, Sigma). DAPI (0.05 mg/mL, Sigma) was used to stain nuclei. Confocal images were obtained using a Leica TCS SP5 II with a HyD detector or an Olympus FV1000 confocal microscope with a GaAsP detector. High resolution confocal images were obtained using a Zeiss LSM 880 with Airyscan super-resolution mode. Three-dimensional confocal reconstruction were performed by Imaris software (Bitplane).

## Results

### The P-element based loss of function screen

To better uncover the molecular mechanisms of BC migration, we performed a P-element based loss-of-function screen using MARCM method. In this screen, we utilized 240 P-element lines with insertions on the chromosomal arm 2L from the UCLA “Bruinfly” FRT-lethal P collection [43]. All the 240 lines are homozygous lethal and a FRT site have been recombined with these P-element insertions [43]. Thus, our screen covers about 240 lethal genes. The number of essential genes present on chromosomal arm 2L is estimated to be about 1000. Therefore, the screen we performed was not saturated. The screen data was collected in S2 Table.

Of the 240 lines we have tested, 25 (~10%) displayed no MARCM clones, suggesting that the corresponding mutations cause cell lethality and may affect genes required for cell viability. Alternatively the FRT site could be absent for various reasons, making recombination no longer possible. Two mutations, *taiman*^*k15101*^ and *Rack1*^*EY128*^, already known to be required for BC migration were identified (S1C and S1E Fig). Taiman is a steroid hormone receptor coactivator and has been reported to be required for BC migration [10]. All of the *taiman*^*k15101*^ mutant clones show BC migration delay (S1I Fig). *Rack1*^*EY128*^ is a null allele of *Rack1*, which encodes a cytoplasmic scaffolding protein with seven WD repeats [44]. We have previously shown *Rack1* is required for BC migration and morphology maintenance [45]. Two mutations not previously described were also uncovered. They are *dlg5*^*KG748*^ and *Scim13*^*1*^. The two mutations show weak phenotype of BC migration defects (S1D, S1F and S1I Fig). About 30% mutant clones of the two mutant lines show BC migration delay (S1I Fig). The P-element in *Scim13*^*1*^ disrupts the gene *CG31689* that encodes an ATP-binding cassette subfamily G protein. We found that *CG31689* is specifically expressed in BC clusters from the analysis of its GFP trap mutant *CB03239* [46] (S1G–S1H” Fig). The mutation *dlg5*^*KG748*^ contains a KG P-element insertion 1158bp upstream of the translation start site of *CG6509*. *CG6509* encodes the *Drosophila* homolog of *dlg5*, a member of MAGUK family. MAGUK family proteins mainly function as scaffolding proteins involved in cell junction assembly, apical-basal polarity, signaling transduction and cell proliferation [47–50]. Dlg5 contains a coiled-coil domain, four PDZ domains, an SH3 domain and a GUK domain. All these modular motifs in Dlg5 are protein-protein interaction domains that are consistent with features of a scaffold protein.

### Dlg5 is required for BC cluster migration and integrity maintenance

**Fig 1:**
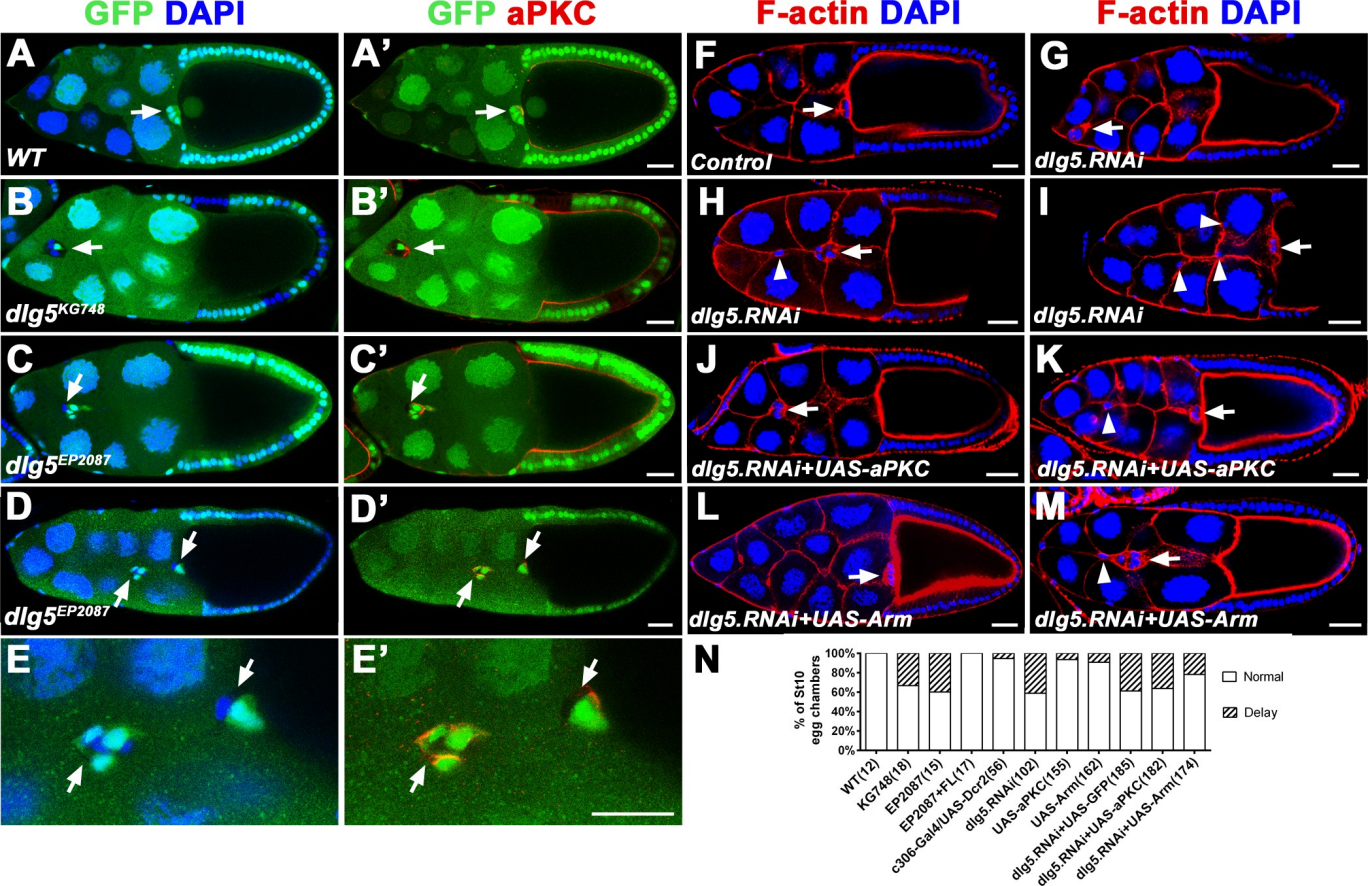
Dlg5 is required for BC cluster migration and integrity maintenance. A, A wild-type BC cluster reaches the border between nurse cells and oocyte in a stage 10 egg chamber. B-C’, *dlg5*^*KG748*^ (B-B’) or *dlg5*^*EP2087*^ (C-C’) mosaic BC cluster fails to migrate to the border at stage 10. D-D’, *dlg5*^*EP2087*^ mosaic BC cluster shows disruption of the cluster integrity. E-E’, A magnified view of the dissociated mosaic cluster as shown in D-D’. F, The *c306-Gal4/UAS-Dcr2* stage 10 egg chamber was shown as control. G-I, Expression of *dlg5*.*RNAi* driven by *c306-Gal4* delayed BC migration (G) and disrupted the cluster integrity (H and I). J-M, The migration delay caused by *dlg5*.*RNAi* was not rescued by overexpression of aPKC (J), but could be partially rescued by overexpression of Arm (L). The dissociated clusters caused by *dlg5*.*RNAi* were not rescued by overexpression either aPKC (K) or Arm (M). BC clusters are marked by arrows. The dissociated or stretched BCs are marked by arrowheads. Stages of the egg chambers were identified by an established method [54]. Stage 9: M; Stage 10a: B, C, G, H, J-L. Stage 10b: A, D, F, I. Scale bars: 20μm.

To validate the *dlg5*^*KG748*^ phenotype observed in the screen, we utilized the other *dlg5* mutation *dlg5*^*EP208*^ to analyze the BC migration phenotype. The lethal mutation *dlg5*^*EP2087*^, which was not covered in this screen, contains an EP P-element insertion 1129bp upstream of the translation start site of *CG6509*. The two P-element mutations, *dlg5*^*KG748*^ and *dlg5*^*EP208*^, failed to complement each other. Our previous work showed both of the two mutants were hypermorphic alleles of *dlg5*, and *dlg5*^*EP208*^ was stronger than *dlg5*^*KG748*^ [37]. We examined the mutant clones of the two alleles in BC clusters using genetic mosaic methods (Flp/FRT). The results showed that mosaic BC clusters containing clone mutant for either *dlg5*^*KG748*^ or *dlg5*^*EP208*^ displayed significant migration delay (Fig 1A–1C’). In wild-type stage 10 egg chambers, all of the BC clusters reached the oocyte border, whereas 33% (*dlg5*^*KG748*^) or 40% (*dlg5*^*EP208*^) of the mutant mosaic BC clusters failed to reach the border by stage 10 (Fig 1A–1C’ and 1N). In some cases, the *dlg5*^*EP208*^ mosaic BC clusters showed disruption of the cluster integrity and two or more parts of the dissociated clusters were found in *dlg5* mutant clones (Fig 1D–1E’). All the phenotypes could be fully rescued by *dlg5* full-length transgene *Ubi-Dlg5*.*EGFP*, which confirmed that defects in BC migration were caused by loss of function of *dlg5* [37]. These data demonstrate that Dlg5 is required for BC migration and cell-cell adhesion in the BC clusters. We also performed RNAi analyses in the BC clusters using *c306-Gal4*. *UAS-Dicer2* was used to enhance the RNAi efficiency [51]. *c306-Gal4* drives expression not only in entire BC clusters but also in anterior and posterior follicle cells from early stage egg chambers [52, 53]. Expressing *dlg5*.*RNAi* in both BCs and PCs using *c306-Gal4* inhibited BC migration in about 40% of stage 10 egg chambers (Fig 1F, 1G and 1N), which was a little stronger than previously reported [35]. Expressing *dlg5*.*RNAi* also caused dissociated clusters (Fig 1H and 1I), which was similar to the phenotype of *dlg5* mutants. Expressing *dlg5*.*RNAi* in the BC clusters by *slbo-Gal4* or *USG-Gal4* (a combination of *upd-Gal4* and *slbo-Gal4*) produced similar migration defects (S2 Fig). All the results indicated that Dlg5 is required not only for BC migration but also for adhesion of BCs to each other and to the PCs.

### Apical-basal polarity of BC clusters

**Fig 2:**
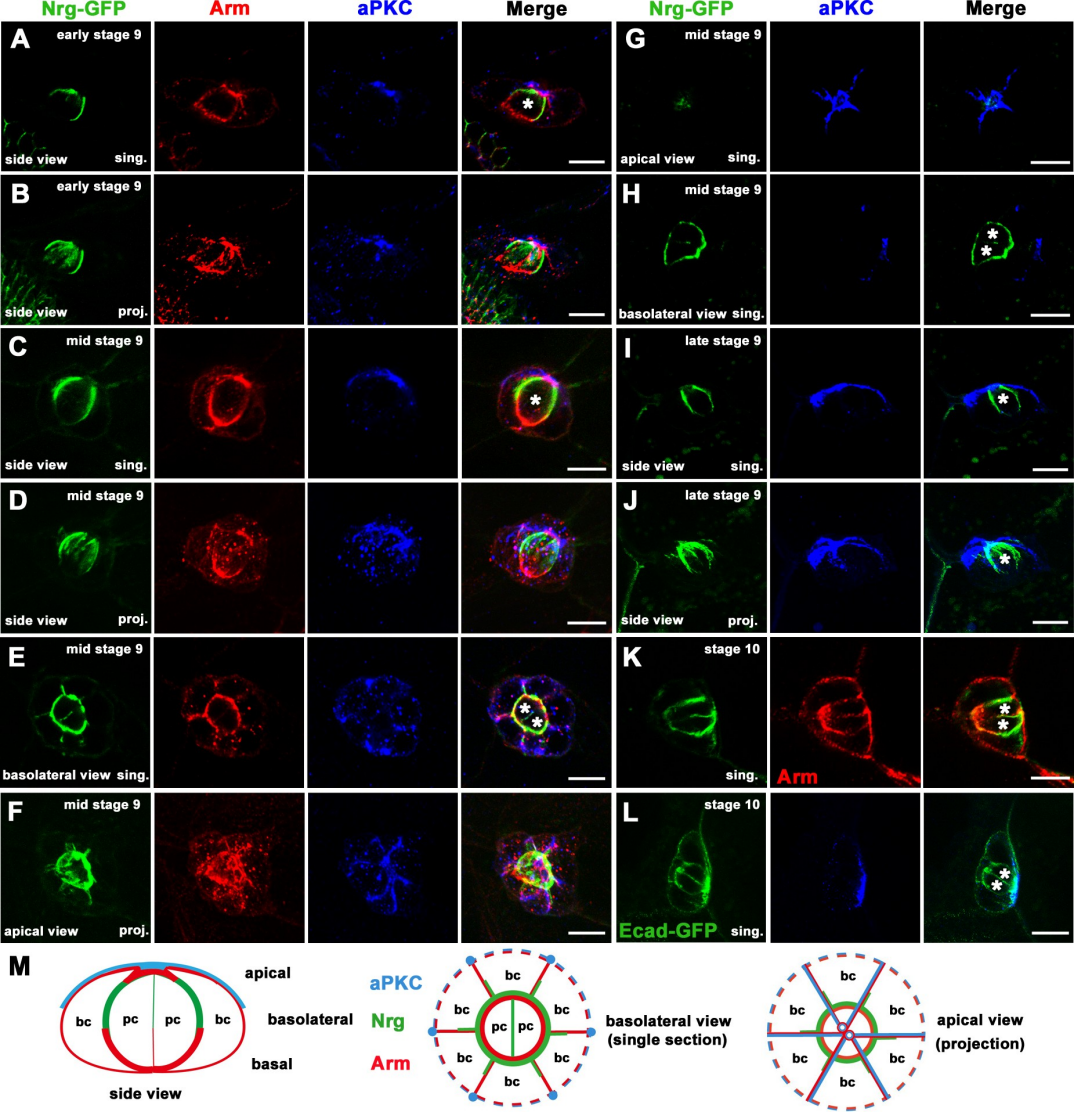
The apical-basal polarity of BC clusters. A-F, Single confocal sections (A, C, E) and corresponding z-series projections (B, D, F) of BC clusters at early stage 9 (A, B) and middle stage 9 (C, F) egg chambers. Side views were shown in A-D. Basolateral views were shown in E. Apical views of z-series projections were shown in F. G and H, Two single confocal sections of the same BC cluster at middle stage 9. Both apical views (G) and basolateral views (H) were shown. I and J, Side views of the BC cluster at late stage 9. Both single sections (I) and z-series projections were shown (J). K and L, Single sections of BC clusters at stage 10. M, Schematics of apical-basal polarity proteins distribution in BC clusters. Side view (left), basolateral view (middle) and apical view (right) were shown. sing., single sections. proj., z-series projections. bc, border cell. pc, polar cell. PCs were indicated by asterisks. Green represents Nrg-GFP from A to K but represents Ecad-GFP in L. Red represents Arm and blue represents aPKC. Scale bars: 10μm.

BC clusters are originally from the anterior follicle epithelium that maintains apical-basal polarity. Turning from epithelial cells to the part-epithelial and part-mesenchymal BCs, their apical-basal polarity is significantly remodeled. Cell-cell adhesions of follicle cells are partially maintained in BC clusters. We have previously shown that Dlg5 regulates the apical polarity of follicle epithelia [37]. In this study, we first examined the apical-basal polarity of wild-type BC clusters in detail. Armadillo (Arm), aPKC and Neuroglian (Nrg) are used as markers for adherens junctions (AJs), apical junctions and septate junctions respectively. At early stage 9, BC clusters detach from the follicle epithelia and invaded the nurse cells [5]. At middle stage 9, BC clusters also undergo rotation during migration. Therefore, both side views and top views (including apical views and basolateral views) of BC clusters were analyzed. In single confocal sections or z-series projections, the asymmetric distribution of aPKC was observed in the BC clusters from early stage 9 to stage 10 (Fig 2A–2J and 2L). At stage 9, aPKC accumulated at the junctions between adjacent BCs and was found at high levels near the apical junction of the BC cluster (Fig 2A–2J). The localization of aPKC was like a claw shape in the projection of confocal z sections of BC clusters (Fig 2F). At stage 10, aPKC were found at high levels at the surface of the BC cluster facing the oocyte (Fig 2L). aPKC were not detected at the contacts between BCs and PCs (Fig 2A–2J and 2L). The adherens junctional protein Arm was detected throughout the lateral junctional surface of the BCs and enriched in both the apical and basal domains of the region between BCs and PCs (Fig 2A–2F and 2K). At some of the apical junctional area, Arm was overlapped with aPKC (Fig 2A–2D). These findings are consistent with the previous studies on the localization of apical proteins Crumbs, Par6 and Baz and the adherens junction component Ecad in the BC cluster [16, 17, 20]. It has been reported that N-cadherin (Ncad) is expressed in follicle cells in early to mid-oogenesis but disappears at stage 10 oogenesis [55]. In our previous study, Ncad is the most significantly reduced protein among polarity proteins in *dlg5* mutant follicle cell clones [37]. However, we found that no Ncad was expressed in BC clusters from early stage 9 to stage 10 since no significant signal was detected by the Ncad antibody in BC clusters (S3 Fig). The septate junctional protein Nrg was detected in the lateral domain of the surfaces between the BCs and the PCs (Fig 2A–2K). From the side view, we found that Nrg-GFP signal complemented with that of Arm (Fig 2A–2D). In the Nrg-GFP localization region, Arm was not enriched (Fig 2A–2D). The schematic diagram in Fig 2M summarizes the expression and distribution of the apical-basal polarity proteins in BC clusters.

### Dlg5’s expression pattern and subcellular localization in BC clusters

To determine the expression pattern and subcellular localization of Dlg5, we utilized three transgenic flies expressing GFP- or TagRFP-T- tagged Dlg5. The genomic construct transgene *Dlg5-TagRFP-T* and the *Ubi-Dlg5*.*EGFP* transgene were previously generated [37]. We have reported that both of the two transgenes are functional and can rescue the lethality of the *dlg5* mutants [37]. The *Ubi-Dlg5*.*TagRFP-T* transgene generated in this study was inserted at the 51C1 attP site on the second chromosome. To perform lethality rescue assay, we generated a chromosome containing both *Ubi-Dlg5*.*TagRFP-T* and *dlg5*^*KG748*^. Flies with this chromosome were able to be kept as a homozygous stock for more than 20 generations and the chromosome also complements with the deficiency chromosome *Df(2L)BSC242* that deletes the *dlg5* locus. This result indicate that the chimera proteins expressed by the construct *Ubi-Dlg5*.*TagRFP-T* can function normally even in the almost complete absence of endogenous Dlg5.

**Fig 3:**
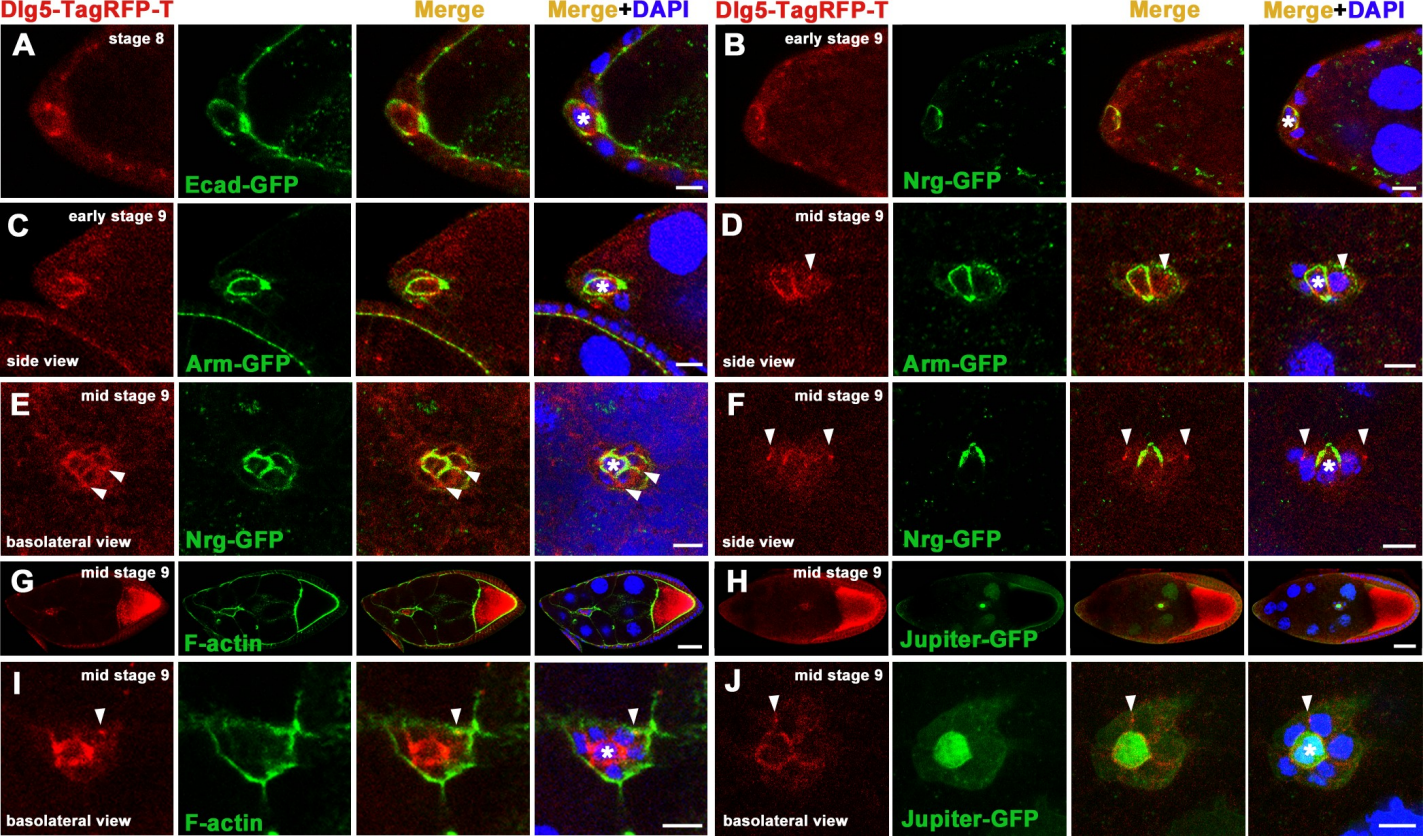
The expression and localization of Dlg5 in BC clusters identified by the genomic transgene *Dlg5-TagRFP-T*. A-F, The expression and localization of Dlg5-TagRFP-T in stage 8 anterior follicle cells (A), and in early stage 9 BC clusters at the initiation of migration (B) or at the invasion of migration (C), and in middle stage 9 BC clusters during migration (D-F). G-J, The expression and localization of Dlg5-TagRFP-T in middle stage 9 BC clusters. The contacts between BCs and nurse cells were marked by F-actin (G, I). The PCs were marked by Jupiter-GFP (H, J). I and J were magnified views of G and H respectively. Dlg5-TagRFP-T mainly localized at the surfaces between BCs and PCs, and also had spot-like localization marked by arrowhead at the contacts between BCs. PCs were indicated by asterisks. Dlg5-TagRFP-T and DAPI was marked by red and blue respectively. F-actin and other GFP fusion proteins were marked by green, which were labelled individually in the green channel. Scale bars: 20μm in G andH and 10μm in others.

First, we utilized the genomic construct *Dlg5-TagRFP-T* for analysis of the expression and localization of Dlg5 in BC clusters. PCs in the BC clusters could be distinguished by Arm-GFP, Ecad-GFP, Nrg-GFP or Jupiter-GFP markers (Fig 3). We found at different stages of BC migration, Dlg5-TagRFP-T mainly localized at the basolateral surfaces between BCs and PCs (Fig 3A–3J). At stage 8 or early stage 9 before invading, Dlg5-TagRFP-T was not colocalized with Ecad-GFP but colocalized with Nrg-GFP around PCs (Fig 3A and 3B). At early stage 9 or mid stage 9 after invading, Dlg5-TagRFP-T was partially colocalized with Arm (Fig 3C and 3D). This distribution pattern was similar to the Nrg localization (Fig 3E and 3F). At mid stage 9, Dlg5-TagRFP-T was also colocalized with Nrg in the contacts between BCs and PCs (Fig 3E and 3F). However, there were also some punctate Dlg5-TagRFP-T localization in the junctional region between BCs (Fig 3D–3F, 3I and 3J), suggesting that Dlg5 might be also involved in cell adhesions between BCs. Dlg5-TagRFP-T was strongly localized around PCs but almost not localized in the contacts between BCs and nurse cells (Fig 3G–3J), suggesting that Dlg5 contributes to the cell-cell adhesion inside BC clusters but not the outside.

**Fig 4:**
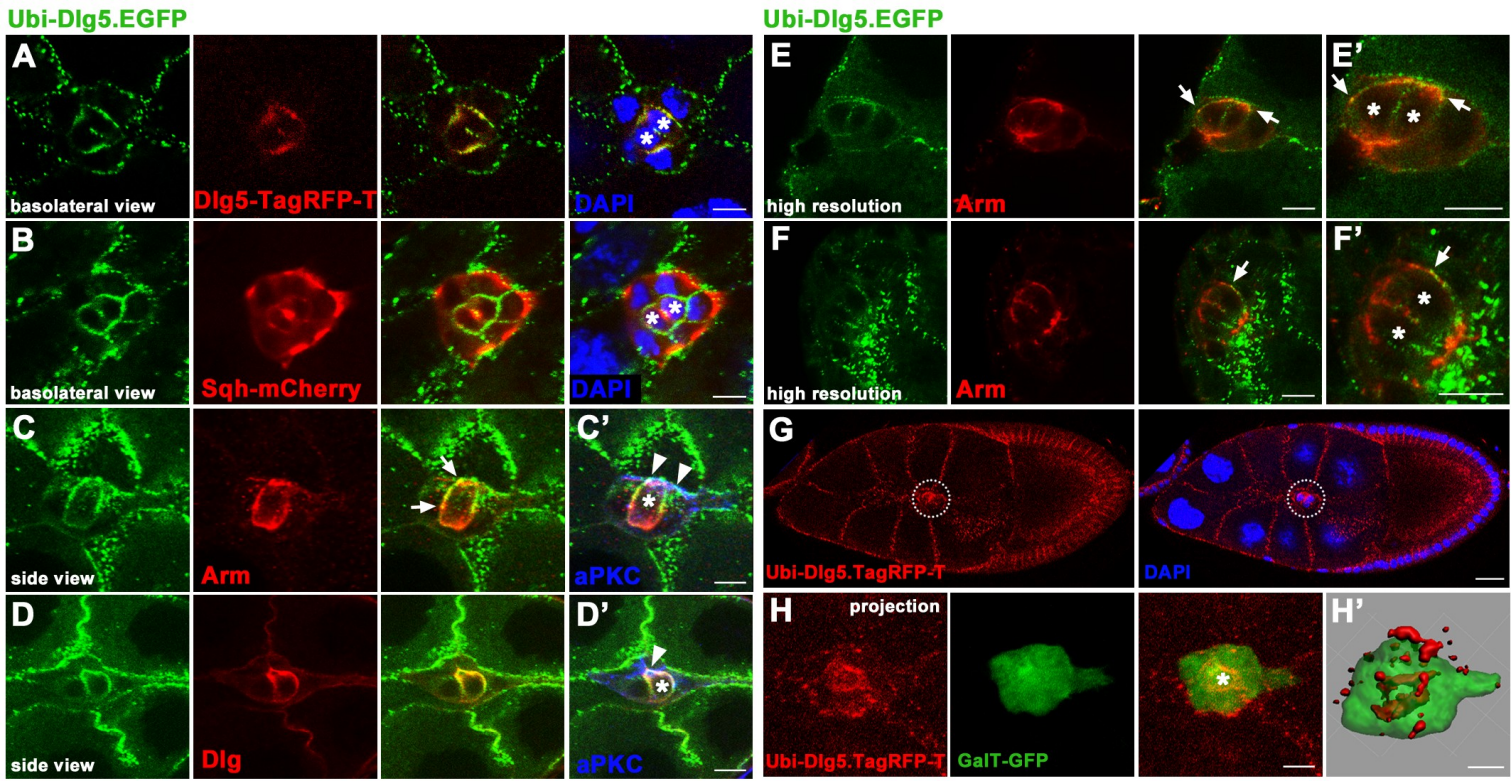
Subcellular localization of Dlg5 in BC clusters identified by *Ubi-Dlg5*.*EGFP* or *Ubi-Dlg5*.*TagRFP-T*. A-D’, Subcellular localization of Dlg5-GFP (green) expressed by *Ubi-Dlg5*.*EGFP* in BC clusters. Dlg5-GFP is colocalized with Dlg5-TagRFP-T expressed by the genomic construct *Dlg5-TagRFP-T* in surfaces between BCs and PCs (A). Dlg5-GFP is colocalized with Dlg (D), partially colocalized with Arm (C) in surfaces between BCs and PCs, but not colocalize with Sqh-mCherry in surfaces between BCs and nurse cells (B). At apical domain of BC clusters, a small region of overlap between Dlg5-GFP (green) and aPKC (blue) was detected (C’, D’). Arrowheads indicate the apical overlap between Dlg5-GFP and aPKC (C’, D’). E-F’, High resolution confocal images obtained using a Zeiss LSM 880 with Airyscan super-resolution mode. Arrows indicate the partial colocalization between Dlg5-RFP and Arm in BC clusters (E-F’). G-H’, Subcellular localization of Dlg5-RFP (red) expressed by *Ubi-Dlg5*.*TagRFP-T* in BC clusters. White dotted circles indicated the BC cluster in G. Dlg5-RFP mainly localized in surfaces between BCs and PCs (G-H’). BC cluster was marked by GalT-GFP (H, H’). Z-series projections (H) and three-dimensional confocal reconstruction (H’) of the BC cluster were shown. The spot-like aggregations of Dlg5-RFP between BCs and nurse cells were not localized in BC clusters (H’). Blue color represents DAPI in A, B and G but represents aPKC in C’ and D’. PCs were indicated by asterisks. Scale bars: 20μm in G and 10μm in others.

Due to a significantly higher level of expression from the *Ubi* transgenes than that from the genomic transgene, we also utilized the *Ubi* transgenes *Ubi-Dlg5*.*EGFP* and *Ubi-Dlg5*.*TagRFP-T* for analysis of Dlg5’s subcellular localization (Fig 4A–4H’). Dlg5-GFP expressed by *Ubi-Dlg5*.*EGFP* is colocalized with Dlg5-TagRFP-T expressed by the genomic construct *Dlg5-TagRFP-T* in BC clusters (Fig 4A), indicating that the subcellular localization of Dlg5-GFP is normal and not altered by *Ubi* promoter. To mimic the endogenous distribution of Dlg5, we expressed Dlg5-GFP in the homozygous *dlg5*^*EP2087*^ animals by the *Ubi-Dlg5*.*EGFP* transgene (Fig 4C–4F’), and expressed Dlg5-RFP in the homozygous *dlg5*^*KG748*^ animals by the *Ubi-Dlg5*.*TagRFP-T* transgene (Fig 4G). The subcellular localization pattern of the *Ubi* expressed Dlg5-GFP was very similar to that of the genomic expressed Dlg5-TagRFP-T, displaying a lateral localization in surfaces between BCs and PCs (Fig 4A–4F’). Both simple confocal images (Fig 4C) and high resolution confocal images (Fig 4E–4F’) showed that Dlg5-GFP was partially colocalized with Arm, and almost completely colocalized with Dlg in BC clusters (Fig 4D). At small region of the apical domain, Dlg5-GFP also overlapped with aPKC (Fig 4C’ and 4D’). There were also punctate Dlg5-GFP localization in junctional region between BCs (Fig 4B). From analysis of the colocalization between *Ubi-Dlg5*.*EGFP* and *Sqh-mCherry* and the three-dimensional reconstructions of *Ubi-Dlg5*.*TagRFP-T* in BC clusters, we found that the strong punctate localization of Dlg5 in surfaces between BCs and nurse cells was due to the strong expression of the *Ubi* transgenes in nurse cells (Fig 4B and 4H’). Dlg5-TagRFP-T expressed by the genomic construct *Dlg5-TagRFP-T* also shows no distribution in contacts between BCs and nurse cells (Fig 4A). We previously reported that Dlg5 colocalized with Arm and apical proteins in follicle cells of early stage egg chambers but colocalized with Dlg in follicle cells of stage 10 egg chambers [37]. The distribution of Dlg5 in BC clusters are similar to its localization pattern in follicle cells of stage 10 egg chambers. Besides Nrg and Dlg, we also found Dlg5 was colocalized with the basolateral proteins ATPα (Na^+^,K^+^-ATPase α subunit) and Lac (Lachesin) (S4 Fig). Taken together, these results indicate that Dlg5 is expressed in BC clusters and mainly localized in surfaces between BCs and PCs with similar localization pattern of septate junctional protein Nrg or Dlg. Both the dissociated cluster phenotype of Dlg5 deficiency and the cell-cell contact localization pattern of Dlg5 in BC clusters suggest that Dlg5 functions in cell adhesions and helps keep BCs together in a collective cluster.

### Dlg5 deficiency affects apical-basal polarity in BC clusters

**Fig 5:**
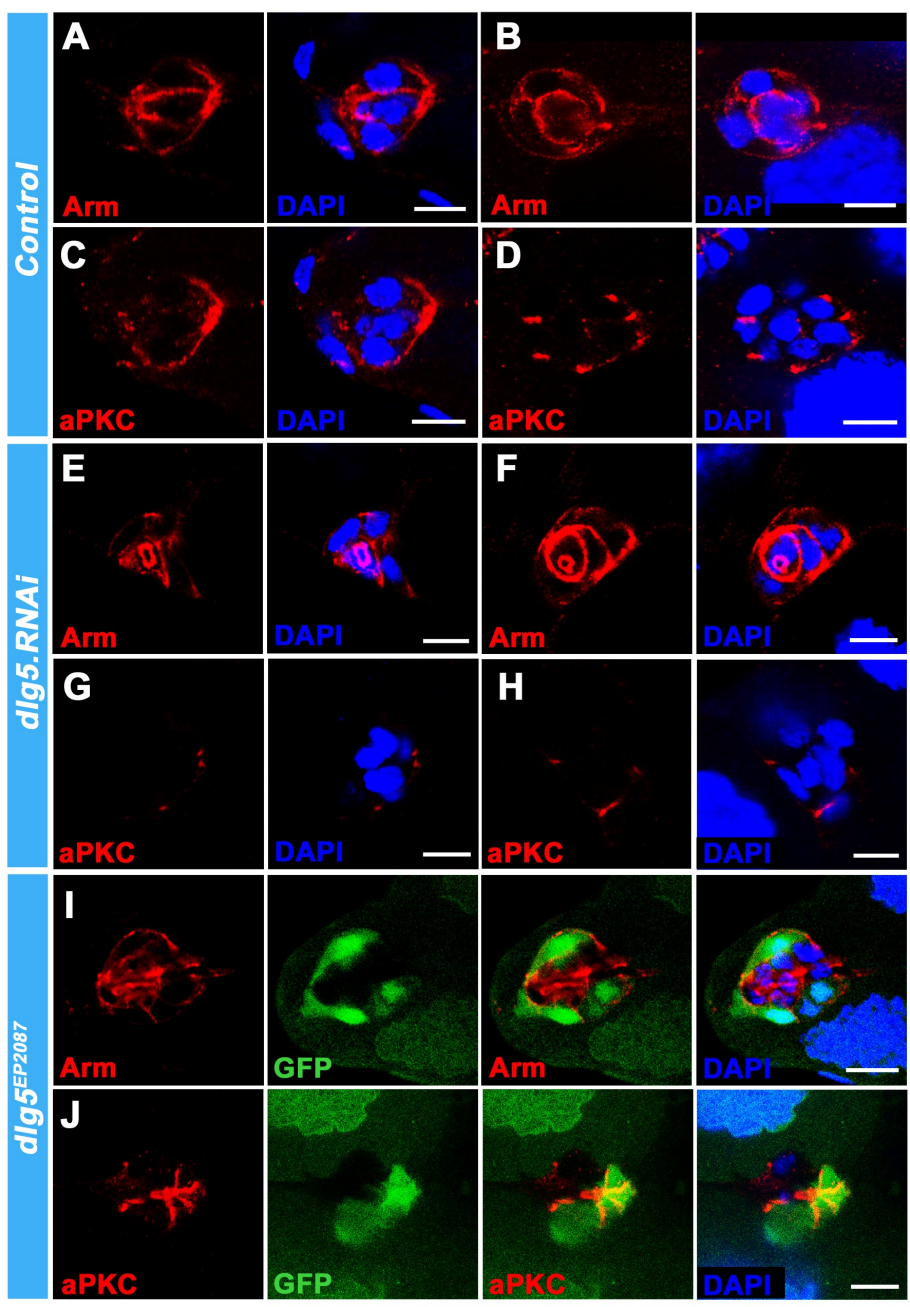
The distributions of Arm and aPKC were specifically affected in Dlg5 deficient BC clusters. A-D, In *c306-Gal4/+* control BC clusters, Arm (A, B) and aPKC (C, D) displayed normal distribution in the invading stage (A, C) and the migrating stage (B, D). E-H, Expression of *dlg5*.*RNAi* driven by *c306-Gal4* resulted in misdistribution of Arm (E, F) and reduction of aPKC (G, H) in both the invading stage (E, G) and the migrating stage (F, H) BC clusters compared with the *c306-Gal4/+* controls (A-D). A-H were single confocal sections. The corresponding z-series projections were shown in S5A–S5H Fig. I and J, In BC clusters, the distribution of Arm was disrupted (I) and the localization of aPKC was reduced (J) in *dlg5*^*EP2087*^ mutant clones marked by the loss of GFP. GFP and DAPI were marked by green and blue respectively. Arm and aPKC were marked by red, and labelled individually in the red channel. Scale bars: 10μm.

Next, we attempted to determine whether apical-basal polarity was affected by Dlg5 deficiency. A previous study using *dlg5 RNAi* did not find gross defects in the localization of adherens junction and polarity proteins [35]. We examined the localization of the apical protein aPKC, the adherens junctional marker Arm and the basolateral markers Dlg and Nrg in *dlg5* mutant clones or *dlg5 RNAi* BCs (Fig 5, S5 Fig and S6 Fig). We found that the distribution of Arm was disrupted in *dlg5 RNAi* BC clusters as compared with the controls (single sections in Fig 5A, 5B, 5E and 5F, corresponding z-series projections in S5A, S5B, S5E and S5F Fig). The apically localized aPKC in BC clusters was strongly reduced in *dlg5* RNAi BC clusters as compared with the controls (single sections in Fig 5C, 5D, 5G and 5H, corresponding z-series projections in S5C, S5D, S5G and S5H Fig). The misdistribution of Arm and reduction of aPKC were also found in *dlg5*^*EP2087*^ mutant clones (Fig 5I and 5J). The aPKC reduction phenotype was more severe in *dlg5* RNAi BC clusters when combined with *dlg5*^*KG748*^ heterozygous background (S5I and S5J Fig). The basolateral markers Dlg and Nrg were not affected in *dlg5 RNAi* BC clusters (S6A–S6D Fig). The basolateral protein Fasciclin 3 (Fas3), which localized at the membrane interface between the two PCs, was also not affected in *dlg5* RNAi BC clusters (S6E and S6F Fig). These data indicate that Dlg5 specifically functions in apical polarity and adherens junctions, and regulates the distribution of aPKC and Arm during BC migration.

### The function and localization of Dlg5 in BC clusters are dependent on Arm

**Fig 6:**
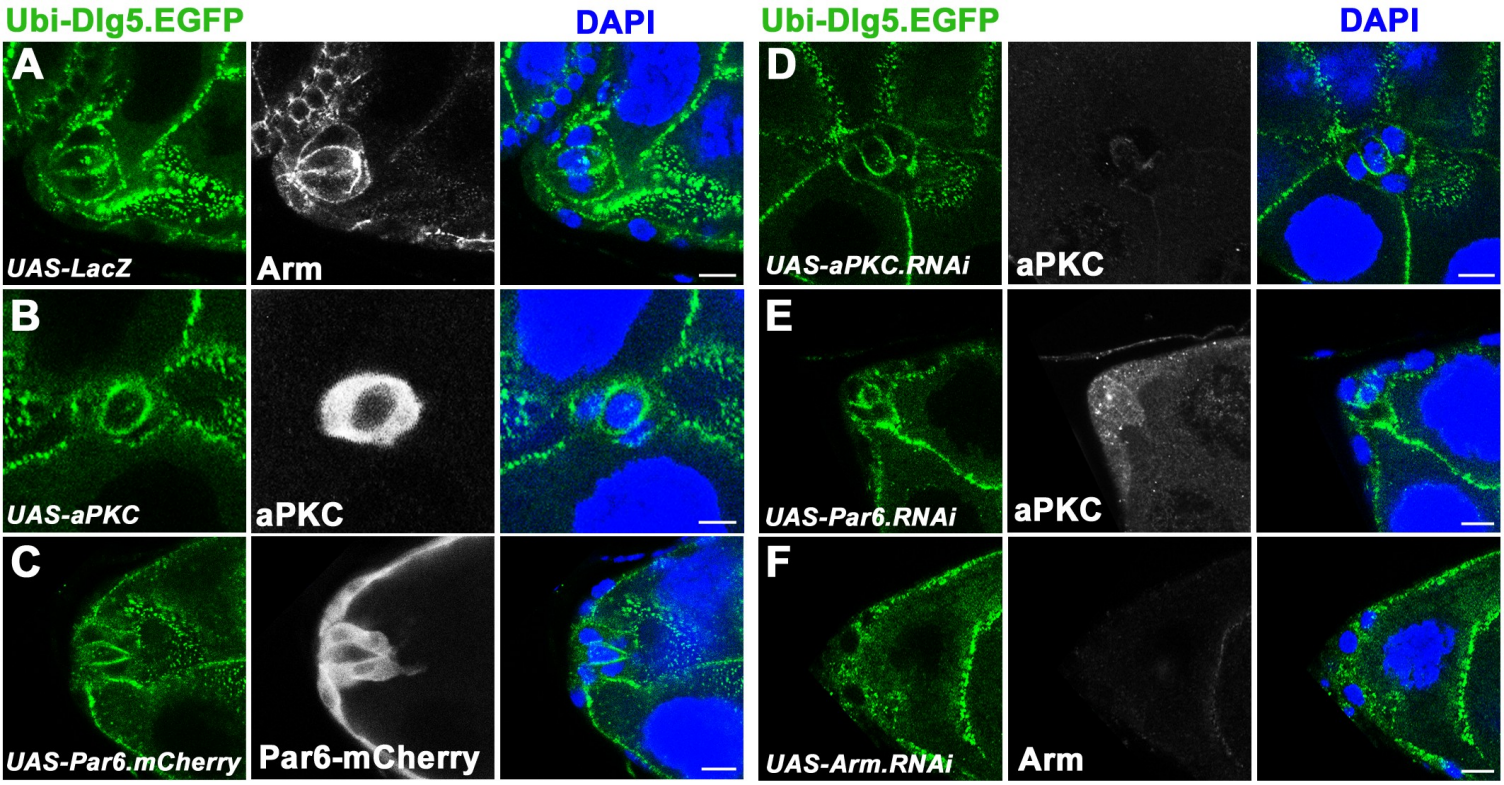
The localization of Dlg5 in BC clusters was dependent on Arm. A, UAS-LacZ control shows lateral membrane localization of Dlg5-GFP expressed by *Ubi-Dlg5*.*EGFP*. B-C, The localization of Dlg5-GFP was not affected by overexpression of aPKC (B) or Par6-mCherry (C). D-E, The localization of Dlg5-GFP was also not affected by RNAi knockdown of aPKC (D) or Par6 (E). F, RNAi knockdown of Arm caused misdistribution of Dlg5-GFP (F). The overexpression or RNAi knockdown was driven by *act5C-GAL4*, *tub-GAL80*^*ts*^ system and induced at 29°C for 3 days at adult stage. The egg chambers of A, C, E, and F are at early stage 9, and the ones of B and D are at middle stage 9. Dlg5-GFP was marked by green. DAPI was blue. Arm, aPKC and Par6-mCherry were marked by white. Scale bars: 10μm.

The immunostaining results demonstrated that distribution of aPKC and Arm were significantly affected in *dlg5* deficient BC clusters (Fig 5). To determine whether they were required for Dlg5’s function in BC migration, we overexpressed aPKC or Arm to perform rescue of the BC migration delay that was resulted from RNAi knockdown of *dlg5*. As mentioned above, expressing *dlg5*.*RNAi* in BC clusters caused ~40% migration delay (Fig 1F, 1G and 1I). We found overexpression of Arm was able to partially rescue the migration delay caused by *dlg5*.*RNAi*, showing a 22% migration delay (Fig 1L and 1I), though the aPKC reduction was not rescued by the overexpression of Arm (S7A, S7B and S7D Fig). Whereas, overexpression of aPKC could not rescue the migration delay caused by *dlg5*.*RNAi*, which showed 36% migration delay (Fig 1J and 1I), and the misdistribution of Arm was also not rescued (S7A, S7C and S7E Fig). This result suggests that Dlg5’s function in BC migration is partially dependent on Arm. However, overexpression of Arm or aPKC could not rescue the dissociated phenotype caused by *dlg5*.*RNAi* (Fig 1K and 1M), suggesting that Arm or aPKC was not sufficient for the cell-cell adhesions in BC clusters. Conversely, to determine whether apical proteins or Arm were required for the distribution of Dlg5 in BC clusters, we overexpressed apical proteins (aPKC and Par6) or performed RNAi knockdown of apical proteins (aPKC and Par6) and Arm in *Ubi-Dlg5*.*EGFP* background. We found that overexpression or RNAi knockdown of aPKC or Par6 were not able to affect Dlg5’s localization as compared with the *UAS-LacZ* control (Fig 6A–6E). Whereas, RNAi knockdown of Arm could apparently affect the membrane localization of Dlg5 in the BC cluster as compared with the *UAS-LacZ* control (Fig 6A and 6F). In addition, the RNAi knockdown efficiency of Arm was validated by antibody staining (S8 Fig). This result suggested that Dlg5 localization in BC clusters is regulated by Arm. Interestingly, we found neither RNAi knockdown of aPKC and Par6 nor knockdown of Arm could affect the apical localization of Dlg5 in follicle cells (S9 Fig), suggesting that the interaction between Dlg5 and Arm is strictly critical in the migratory BC cells but not in epithelial cells.

### Structure-function analysis

**Fig 7:**
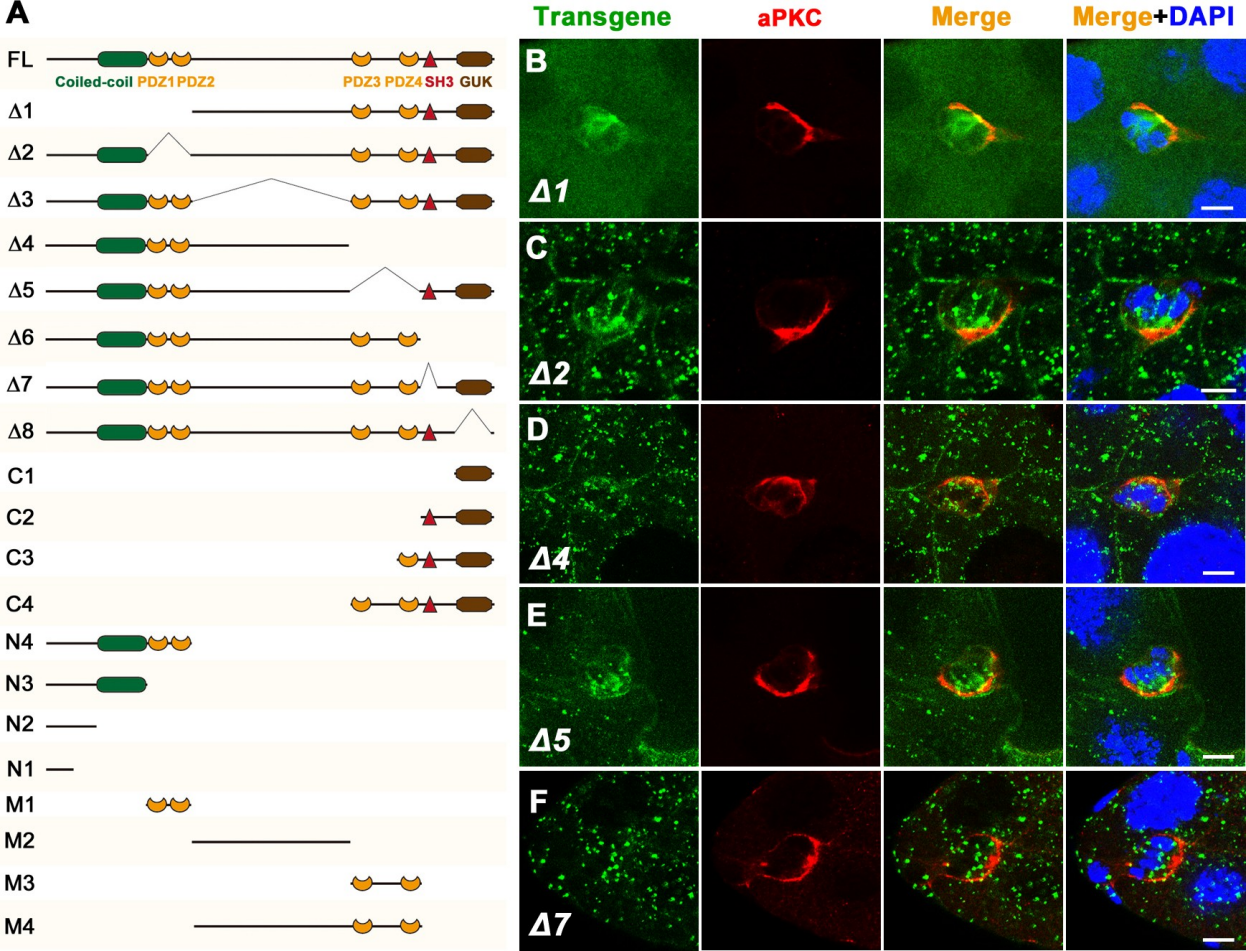
Subcellular localization of various Dlg5 truncated proteins in BC clusters. A, Schematics of various truncated forms of Dlg5. Detailed description of these transgenes was reported previously [37]. B-F, Subcellular localization of Dlg5 full-length (FL) and deletions (Δ1, Δ2, Δ4, Δ5 and Δ7) in BC clusters (green). aPKC was shown in the second column (red). Summary of the Dlg5 deletions’ localization was shown in S3 Table. Subcellular localization of the other Dlg5 truncated transgenes was shown in S10 Fig and summarized in S4 Table. Scale bars: 10μm.

*Drosophila* Dlg5 belongs to the MAGUK superfamily and contains a coiled-coil motif, four PDZ domains, an SH3 domain, and a GUK domain (Fig 7A). To clarify the subcellular localization ability and function of these domains in BC clusters, we utilized a series of *dlg5* transgenes that were previously generated to analyze the subcellular localization and the BC migration delay rescue ability of these transgenes [37]. These *dlg5* transgenes were inserted in the same attP site and expressed a full set of different Dlg5 fragments or truncations (Fig 7A). We first analyzed the subcellular localization ability of these transgenes in BC clusters (Fig 7 and S10 Fig). Consistent with the aforementioned subcellular localization of Dlg5 in BCs, the full length Dlg5-GFP (FL) was localized to the lateral surfaces between BCs and PCs (S10A Fig). Systematically deleting domains or fragments of Dlg5 revealed that the deletion of the C-terminal region (Δ4, deleting C-terminal PDZ3-PDZ4-SH3-GUK region) and the deletion of the SH3 domain (Δ7) or the GUK domain (Δ8) caused complete loss of BC-PC surface localization (Fig 7D and 7F and S10D Fig). On the contrary, expression of the C-terminal MAGUK core domains (C3, PDZ4-SH3-GUK) showed strong uniform membrane targeting including the entire BC-PC surfaces (S10G Fig). Moreover, deletions of the N-terminus (Δ1, deleting the N-terminal Coiled-coil-PDZ1-PDZ2 region), PDZ domains (Δ2 and Δ5, deleting PDZ1-PDZ2 or PDZ3-PDZ4 region respectively), or the linker region (Δ3) could not completely disrupted the BC-PC surfaces localization (Fig 7B, 7C and 7E and S10B Fig). These results suggested that the MAGUK core tandem domains PDZ4-SH3-GUK are required for the BC-PC surface localization. The coiled-coil domain of Dlg5 is a unique feature that distinguishes it from all other Dlg family members. Expression of N-terminal N3 (Coiled-coil) fragment displayed uniform BC-PC surfaces localization (S10J Fig), suggesting the N-terminal coiled-coil domain is important for Dlg5’s cell surface localization. The middle M2 (the linker region) and M4 (Linker-PDZ3-PDZ4) fragments were specifically localized to the BC-PC or BC-BC apical surfaces and colocalized with Arm (S10N and S10P Fig), suggesting that the linker region contributes to the specific localization of Dlg5 in BC-PC surfaces where Dlg5 partially colocalized with Arm. It has been reported that the emerging function of PDZ domain of MAGUK proteins is to bind the extreme carboxy-terminal cytoplasmic tail of transmembrane proteins [47]. However, we found that specific expression of PDZ1-PDZ2 domains (M1) or PDZ3-PDZ4 domains (M3) exhibited a diffused and cytoplasmic distribution pattern lacking any cell membrane localization (S10M and S10O Fig), indicating that the PDZ domains contain no membrane targeting function on their own. Compared to the M1 localization, M3 showed PC enrichment (S10M and S10O Fig), suggesting that PDZ3-PDZ4 but not PDZ1-PDZ2 contributes to BC-PC surfaces enrichment of Dlg5. Compared to the localization of FL, the deletion of PDZ1-PDZ2 (Δ2) or PDZ3-PDZ4 (Δ5) showed strong punctate aggregation in cytosol (Fig 7C and 7E), which might be due to the disruption of the association between Dlg5 and membrane proteins when PDZ domains were absent. Among the deleting fragments of Dlg5, only the deletion of N-terminal Coiled-coil-PDZ1-PDZ2 (Δ1) and the deletion of C-terminal SH3-GUK (Δ6) displayed no punctate aggregation in cytosol or cell surfaces (Fig 7B, S10C Fig), suggesting that both the N-terminal region and the C-terminal region contribute to the aggregation of Dlg5. This result also indicated a possibility that Dlg5 molecules interact with each other in a head-to-tail manner and form large protein complexes that can scaffold and stabilize polarity proteins at the cell surfaces.

**Fig 8:**
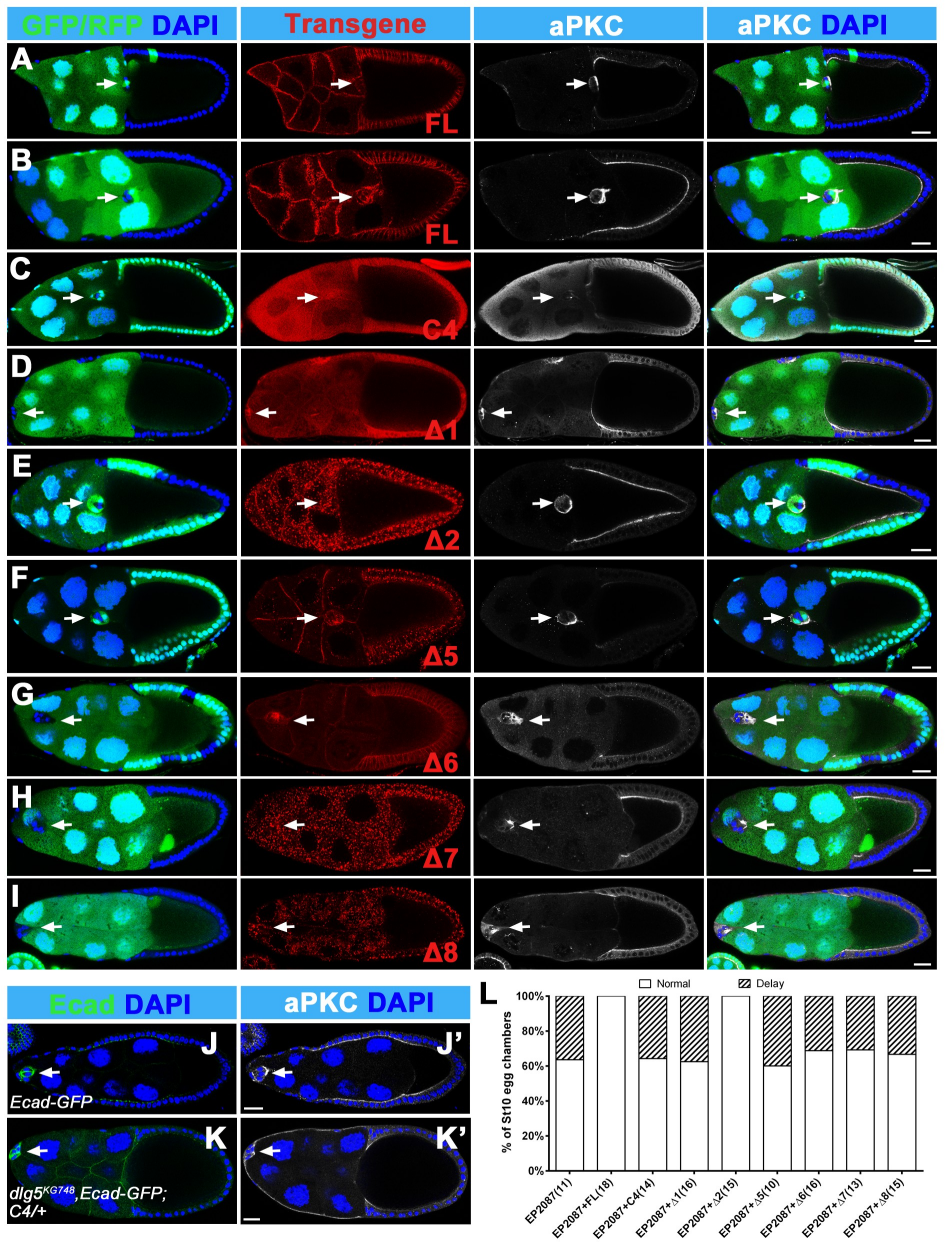
Ability of various truncated forms of Dlg5 to rescue the BC migration delay in *dlg5*^*EP2087*^ mutant mosaic clones. A-I, Confocal images showing different Dlg5 truncation forms rescuing BC migration delay in *dlg5*^*EP2087*^ mutant mosaic clones. The full-length (FL) (A, B) and Δ2 (E) fully rescued the BC migration delay. Whereas C4, Δ1, Δ5, Δ6, Δ7 and Δ8 had no or little rescue ability (C, D, F-I). Mutant clones were marked by the absence of the GFP or RFP (green). Transgenes and aPKC were marked by red and white respectively. J-K’, Escapers from *dlg5*^*KG748*^ homozygous rescued by C4 showed completely no migration of BC clusters and misdistribution of Ecad-GFP (green, K) and reduction of aPKC (white, K’) compared to the wild-type Ecad-GFP (J, J’). L, Quantification of BC migration delay in different genotypes. EP2087, *dlg5*^*EP2087*^. DAPI was marked by blue. Scale bars: 20μm.

We next determined which domains or regions were required for the Dlg5’s function in BC migration. We utilized the truncated transgenes to rescue the migration delay phenotype of BC clusters in *dlg5* mutant clones (Fig 8). The full-length transgene (FL) could fully rescue the migration delay of *dlg5* mutant clones (Fig 8A and 8B). Besides the FL, we found that only the deletion of the PDZ1-PDZ2 region (Δ2) was able to fully rescue the migration delay of *dlg5* mutant mosaic clones (Fig 8E and 8L), suggesting the PDZ1-PDZ2 domains are not required for Dlg5’s function in BC migration. Deletions of the other regions, such as the Coiled-coil deletion (Δ1), the PDZ3-PDZ4 deletion (Δ5), the SH3-GUK deletion (Δ6), the SH3 or GUK specific deletions (Δ7 and Δ8), all failed to rescue the BC migration delay of *dlg5*^*EP2087*^ mosaic clones (Fig 8D and 8F–8I), indicating that both N-terminal coiled-coil domain and the C-terminal PDZ3-PDZ4-SH3-GUK domains are required for BC migration. Expression of the C-terminal fragment, C4 (including PDZ3-PDZ4-SH3-GUK), also failed to rescue the BC migration delay (Fig 8C). We previously showed that the expression of C4 was not able to rescue the lethality of *dlg5*^*EP2087*^, but could partially rescue the lethality of *dlg5*^*KG748*^ [37]. From the escapers of the *dlg5*^*KG748*^*/dlg5*^*KG748*^*; C4/+* flies, we found the egg chambers developed to stage 10 with normal morphology, but all the BC clusters stayed at the anterior of stage 10 egg chambers with no significant migration (Fig 8J–8K’). Immunostaining of the egg chambers showed misdistribution of Ecad and apparent reduction of aPKC. This result indicates that the C-terminal PDZ3-PDZ4-SH3-GUK region cannot replace Dlg5’s function in the migration and the polarity maintenance of BC clusters.

## Discussion

Our study demonstrated that Dlg5 is required for BC migration and the maintenance of apical-basal polarity of BC clusters, since *dlg5* deficiency caused BC migration delay (Fig 1A–1I), reduction of apical polarity protein aPKC, and misdistribution of Arm (Fig 5). We previously shown aPKC is a key polarity determinant in coordinating the function of three distinct cell polarities (apical-basal polarity, front-back polarity and inner-outer polarity) in BC migration [37]. Dlg5 functions as a regulator in BC migration probably through regulating the key polarity determinant protein aPKC in BC clusters [20]. However, the distribution of Dlg5 is affected by RNAi knockdown by Arm but not aPKC (Fig 7), indicating that the localization of Dlg5 is dependent on Arm. We examined the apical-basal polarity of BC clusters in detail (Fig 2), and described the expression and subcellular localization of Dlg5 in BC clusters for the first time (Fig 3 and Fig 4). We found Dlg5 colocalized with septate junctional proteins and mainly localized at the basolateral surfaces between BCs and PCs. Dlg5 also localized at cell-cell contacts between BCs in a punctate pattern, partially overlapping with the adherens junctional protein Arm and the apical protein aPKC. This result suggested that Dlg5 may act as a scaffold and function in the stabilization of apical polarity components including aPKC in the apical junction, as consistent with its roles in the follicle epithelium [37]. The distribution of Dlg5 in BC clusters was different from that in follicle cells of early stage egg chambers but similar to that in follicle cells of stage 10 egg chambers [37]. Both the dissociated phenotype of *dlg5* deficiency and the BC-PC surfaces enrichment of Dlg5 protein suggested that Dlg5 functions in cell-cell adhesions in BC clusters. Furthermore, the distribution of Dlg5 in BC clusters was affected by the RNAi knockdown of Arm but not aPKC or Par6, indicating that the distribution of Dlg5 in BC clusters is regulated by Arm but not apical proteins.

As a MAGUK family member, Dlg5 has many protein-protein interaction domains, such as the Coiled-coil domain, the PDZ domain, the SH3 domain, and the GUK domain that has no catalytic activity [50]. Previous works have determined which domains of mammalian Dlg5 physically interact with junctional and membrane-bound proteins like β-catenin, vinexin, smoothened, and trafficking regulators syntaxin 4 *in vitro* or in cultured cells [27, 29, 33]. We previously reported that the PDZ3-PDZ4 domains of Dlg5 were critical for its function in the follicle cells [37]. In this study, we found that both the N-terminal Coiled-coil domain and the C-terminal PDZ3-PDZ4-SH3-GUK domains were required for Dlg5’s function in BC migration, and the PDZ1-PDZ2 domains were not necessary for BC migration. The PDZ1-PDZ2 domains might have redundant function with the PDZ3-PDZ4 domains. Lastly, similar to the previous study in follicle cells, we found the middle linker region and the MAGUK core domains (PDZ-SH3-GUK) could be individually targeted to the AJs and the membranes respectively in BC clusters.

The role of human DLG5 in cancer proliferation, migration, and cancer development has been reported [28, 56, 57]. Human DLG5 may suppress cell proliferation via interacting with p55 [28]. DLG5 is involved in pancreatic caricinogenesis by suppressing the growth of pancreatic ductal adenocatcinoma (PDAC) [56]. A genome wide RNAi screen showed that DLG5 contributes to invasion and metastasis in breast cancer cell [57]. These studies revealed that DLG5 is a potential molecular target for tumor therapy. In this work, we showed that *Drosophila* Dlg5 promotes collective cell migration by regulating the cluster polarity, providing clues to DLG5’s roles in cancer metastasis.

In conclusion, we found the MAGUK family gene *dlg5* (*discs large 5*) regulates the apical-basal polarity and cluster integrity during border cell migration. Dlg5 is enriched in lateral surfaces between border cells and central polar cells but also shows punctate localization between border cells. The localization of Dlg5 is regulated by Arm. We found that the N-terminal Coiled-coil domain and the C-terminal PDZ3-PDZ4-SH3-GUK domains but not the PDZ1-PDZ2 domains of Dlg5 are required for border cell migration. The Coiled-coil domain and the PDZ4-SH3-GUK domains are critical for Dlg5’s cell surface localization in border cell clusters.

## Supporting information

S1 Fig The P-element screen result. A, Schematic diagram of an egg chamber during BC migration. Left, the BC cluster initiates and invades into the nurse cells at early stage 9. Middle, the BC cluster migrates between nurse cells at middle stage 9. Right, the BC cluster reaches the border between nurse cells and oocyte at stage 10. B, A wild-type BC cluster clones reaches the border between nurse cells and oocyte in a stage 10 egg chamber. C-F, BC migration is delayed in P-element mutants, including *tainman*^*k15101*^ (C), *Scim13*^*1*^ (D), *Rack1*^*EY128*^ (E) and *dlg5*^*KG748*^ (F). Green, GFP. Mutant clones are marked by GFP. G-H”, The GFP expression pattern of *CG31689*^*CB03239*^ in ovaries. *CG31689* specifically expresses in BCs at both stage 9 (G-G”) and stage 10 (H-H”) egg chambers. I, Quantification of the BC migration delay. BC clusters are marked by arrows. Scale bars: 20μm.
(TIF)

S2 Fig Dlg5 knockdown inhibits BC migration. A, The *UAS-Dcr2/+; slbo-Gal4*,*UAS-GFP/+* stage 10 egg chamber was shown as control. B-C’, Expression of *dlg5*.*RNAi* driven by *slbo-Gal4* delayed BC migration (B) and in some case disrupted the cluster integrity (C-C’). D, The *USG-Gal4*, *UAS-Dcr2* stage 10 egg chamber was shown as control. E-F’, Expression of *dlg5*.*RNAi* driven by *USG-Gal4* delayed BC migration (E) and in some case caused dissociated or stretched BC clusters (C-C’). L, Quantification of BC migration delay showed above. BC clusters are indicated by arrows. The dissociated or stretched BCs are indicated by arrowheads. Scale bars: 20μm.
(TIF)

S3 Fig Ncad is not expressed in BC clusters. A-C, In wild-type egg chambers, Ncad is not expressed in BC clusters in early stage 9 (A), middle stage 9 (B) and stage 10 (C). Ncad is expressed in early stage follicle cells (A-C) and in stage 9 follicle cells (A-B), but not expressed in stage 10 follicle cells (C). Arrows indicated the BC clusters. Scale bars: 50μm.
(TIF)

S4 Fig Dlg5 colocalizes with the septate junction proteins in BC clusters. A, Dlg5-RFP (red) expressed by *Dlg5-TagRFP-T* colocalized with ATPα-GFP (green) in the BC cluster. B, Dlg5.TagRFP-T (red) expressed by *Ubi-Dlg5*.*TagRFP-T* partially colocalized with Lac-GFP (green). Scale bars: 10μm.
(TIF)

S5 Fig The localization of Arm and aPKC were affected in Dlg5 deficient BC clusters. A-H, z-series projections of BC clusters corresponding to the single sections of Fig 5A–5H. A-D, In *c306-Gal4/+* control BC clusters, Arm (A, B) and aPKC (C, D) displayed normal distribution in the invading stage (A, C) and the migrating stage (B, D). E-H, Expression of *dlg5*.*RNAi* driven by *c306-Gal4* resulted in misdistribution of Arm (E, F) and reduction of aPKC (G, H) in both the invading stage (E, G) and the migrating stage (F, H) BC clusters compared with the *c306-Gal4/+* controls (A-D). I and J, The phenotypes were more severe in *dlg5* RNAi BCs combined with *dlg5*^*KG748*^ heterozygous background. Especially, the localization of aPKC was almost completely lost (J). DAPI was marked by blue. Arm and aPKC were marked by red, and labelled individually in the red channel. Scale bars: 10μm.
(TIF)

S6 Fig The localization of septate proteins were not affected in Dlg5 deficient BC clusters. A and B, The localization of Dlg (red) was not affected in *dlg5*.*RNAi* BC cluster (B) compared to the control (A). C and D, The localization of Fas3 (red) was not affected in *dlg5*.*RNAi* BC cluster (D) compared to the control (C). E and F, The localization of Nrg-GFP (red) was not affected in *dlg5*.*RNAi* BC cluster (F) compared to the control (E). DAPI was marked by blue. Scale bars: 10μm.
(TIF)

S7 Fig Overexpression of aPKC or Arm can not restore the localization disruption of Arm and aPKC in Dlg5 deficient BC clusters. A, UAS-LacZ control shows normal localization of aPKC (green) and Arm (red). B, Overexpression of Arm causes strong enrichment of Arm in membrane and cytosol. C, Overexpression of aPKC causes strong enrichment of aPKC in the whole cell. D, The reduction of aPKC caused by *dlg5*.*RNAi* could not be rescued by overexpression of Arm. E, The misdistribution of Arm caused by *dlg5*.*RNAi* could not be rescued by overexpression of aPKC. DAPI is marked by blue. Scale bars: 10μm.
(TIF)

S8 Fig *UAS-Arm*.*RNAi* can efficiently knockdown the expression of Arm. A, *UAS-LacZ* control shows normal expression of Arm. B and C, Efficient knockdown of Arm expression using *UAS-Arm*.*RNAi*. Scale bars: 50μm.
(TIF)

S9 Fig Dlg5’s localization in follicle epithelial cells is not affected by RNAi knockdown of apical proteins or Arm. A, *act5C-Gal4*, *tub-Gal80*^*ts*^ control shows apical enrichment of Dlg5.GFP expressed by *Ubi-Dlg5*.*EGFP* in follicle cells. B-D, the distribution of Dlg5.GFP is not affected by RNAi knockdown of aPKC (B), Par6 (C) or Arm (D) in follicle cells. Scale bars: 10μm.
(TIF)

S10 Fig Subcellular localization of various Dlg5 truncated proteins in BC clusters. A-P, Subcellular localization of Dlg5 truncated proteins (FL, Δ3, Δ6, Δ8, C1, C2, C3, C4, N4, N3, N2, N1, M1, M2, M3, M4) in BC clusters (green). Schematics of these truncated forms were shown in Fig 8. Co-staining of polarity markers, Arm or Dlg, was shown in the second column (red). Summary of these Dlg5 truncated transgenes’ localization was shown in S3 and S4 Tables. DAPI is marked by blue. Scale bars: 10μm.
(TIF)

S1 Table Genotypes used in this study. (DOCX)

S2 Table The P-element loss-of-function screen data. (XLSX)

S3 Table Summary of the Dlg5 deletions analyses. (DOCX)

S4 Table Summary of the Dlg5 domains analyses. (DOCX)
