# Supplementary figures and images for: The polarity protein Dlg5 regulates collective cell migration during Drosophila oogenesis
Source: PLoS One. 2019 Dec 19;14(12):e0226061. doi: 10.1371/journal.pone.0226061 (PMC6922378; doi:10.1371/journal.pone.0226061)

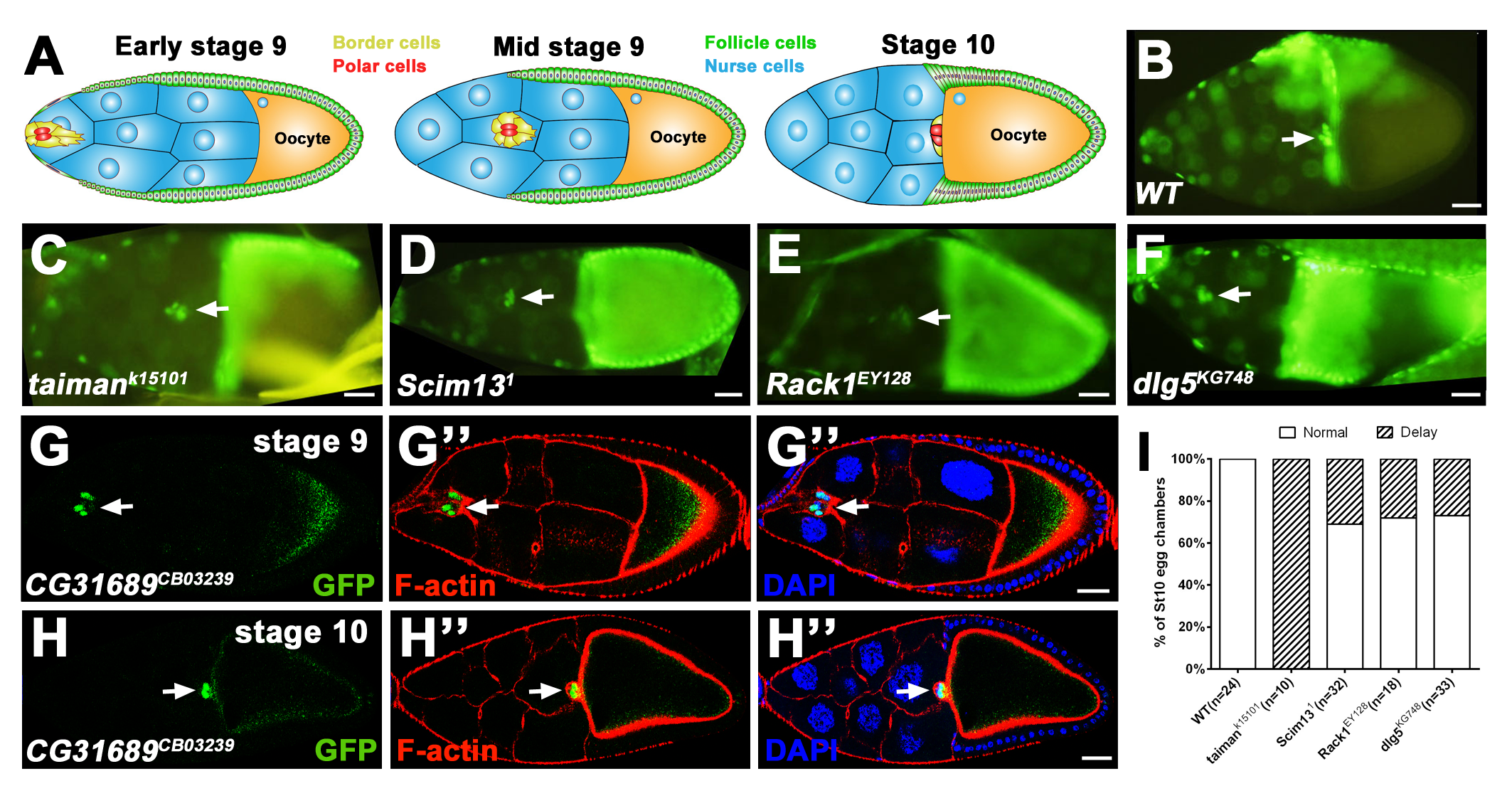

Supplement: S1 Fig — A, Schematic diagram of an egg chamber during BC migration. Left, the BC cluster initiates and invades into the nurse cells at early stage 9. Middle, the BC cluster migrates between nurse cells at middle stage 9. Right, the BC cluster reaches the border between nurse cells and oocyte at stage 10. B, A wild-type BC cluster clones reaches the border between nurse cells and oocyte in a stage 10 egg chamber. C-F, BC migration is delayed in P-element mutants, including tainmank15101 (C), Scim131 (D), Rack1EY128 (E) and dlg5KG748 (F). Green, GFP. Mutant clones are marked by GFP. G-H”, The GFP expression pattern of CG31689CB03239 in ovaries. CG31689 specifically expresses in BCs at both stage 9 (G-G”) and stage 10 (H-H”) egg chambers. I, Quantification of the BC migration delay. BC clusters are marked by arrows. Scale bars: 20μm. (TIF) [file pone.0226061.s001.tif]

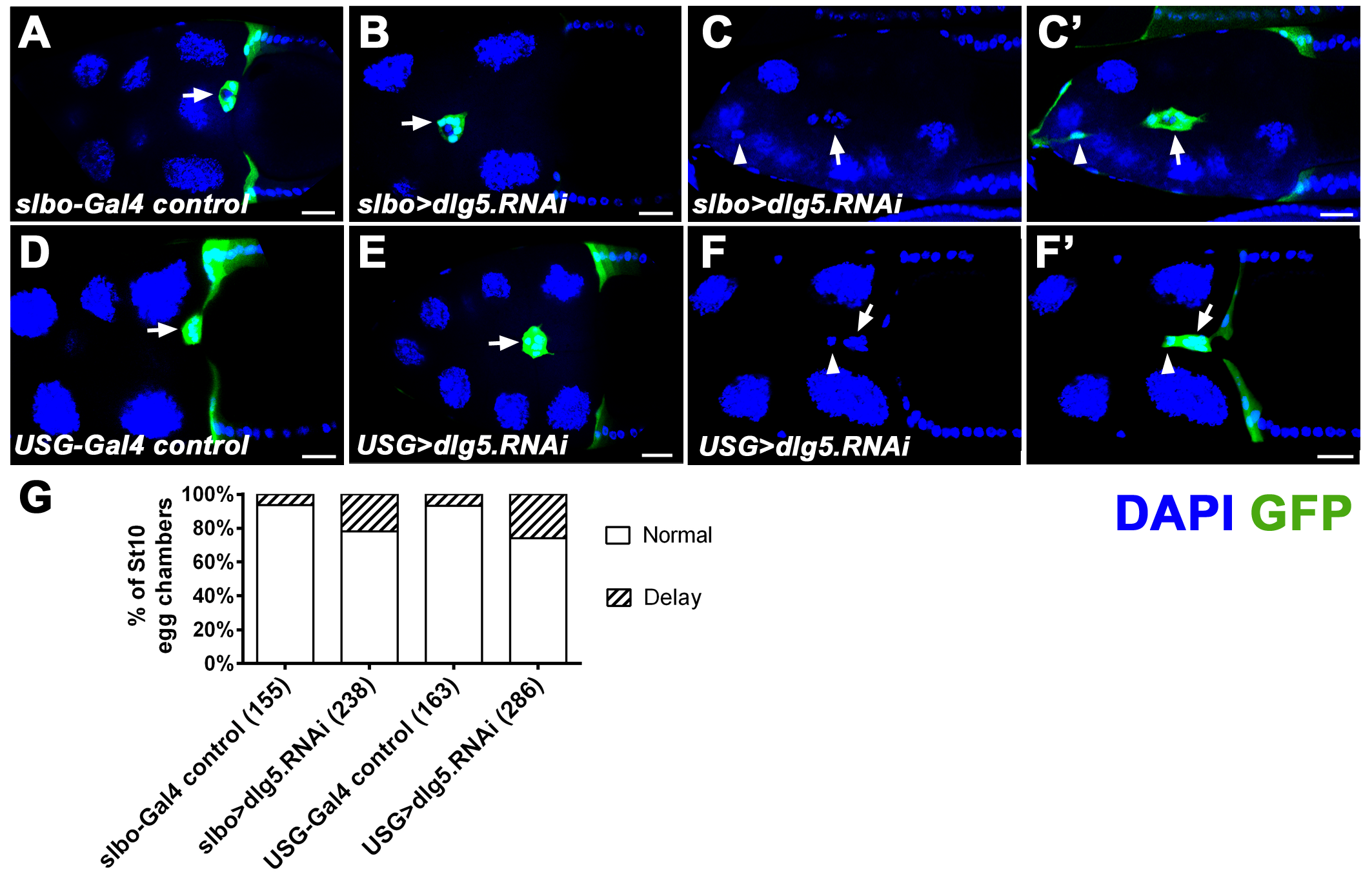

Supplement: S2 Fig — A, The UAS-Dcr2/+; slbo-Gal4,UAS-GFP/+ stage 10 egg chamber was shown as control. B-C’, Expression of dlg5.RNAi driven by slbo-Gal4 delayed BC migration (B) and in some case disrupted the cluster integrity (C-C’). D, The USG-Gal4, UAS-Dcr2 stage 10 egg chamber was shown as control. E-F’, Expression of dlg5.RNAi driven by USG-Gal4 delayed BC migration (E) and in some case caused dissociated or stretched BC clusters (C-C’). L, Quantification of BC migration delay showed above. BC clusters are indicated by arrows. The dissociated or stretched BCs are indicated by arrowheads. Scale bars: 20μm. (TIF) [file pone.0226061.s002.tif]

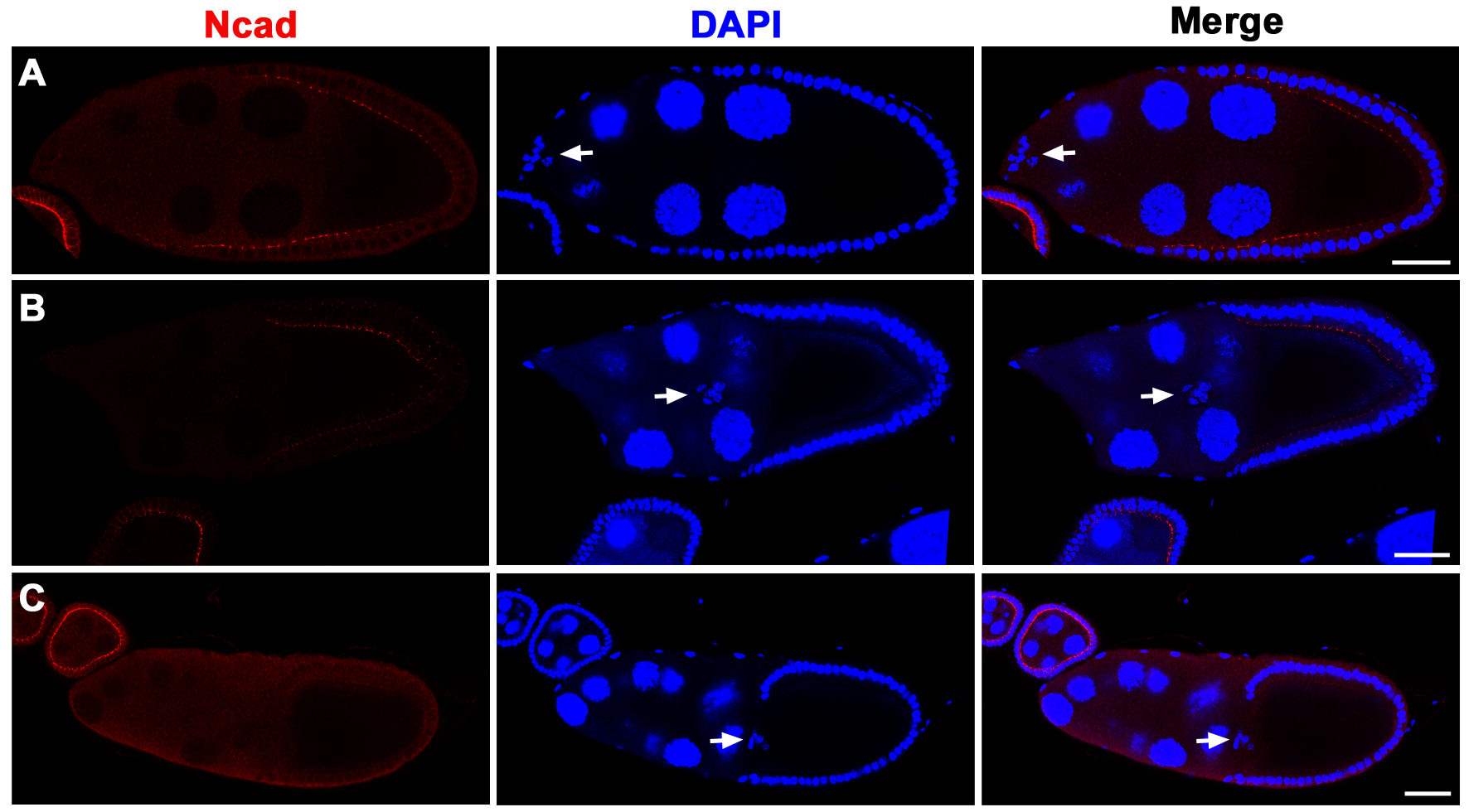

Supplement: S3 Fig — A-C, In wild-type egg chambers, Ncad is not expressed in BC clusters in early stage 9 (A), middle stage 9 (B) and stage 10 (C). Ncad is expressed in early stage follicle cells (A-C) and in stage 9 follicle cells (A-B), but not expressed in stage 10 follicle cells (C). Arrows indicated the BC clusters. Scale bars: 50μm. (TIF) [file pone.0226061.s003.tif]

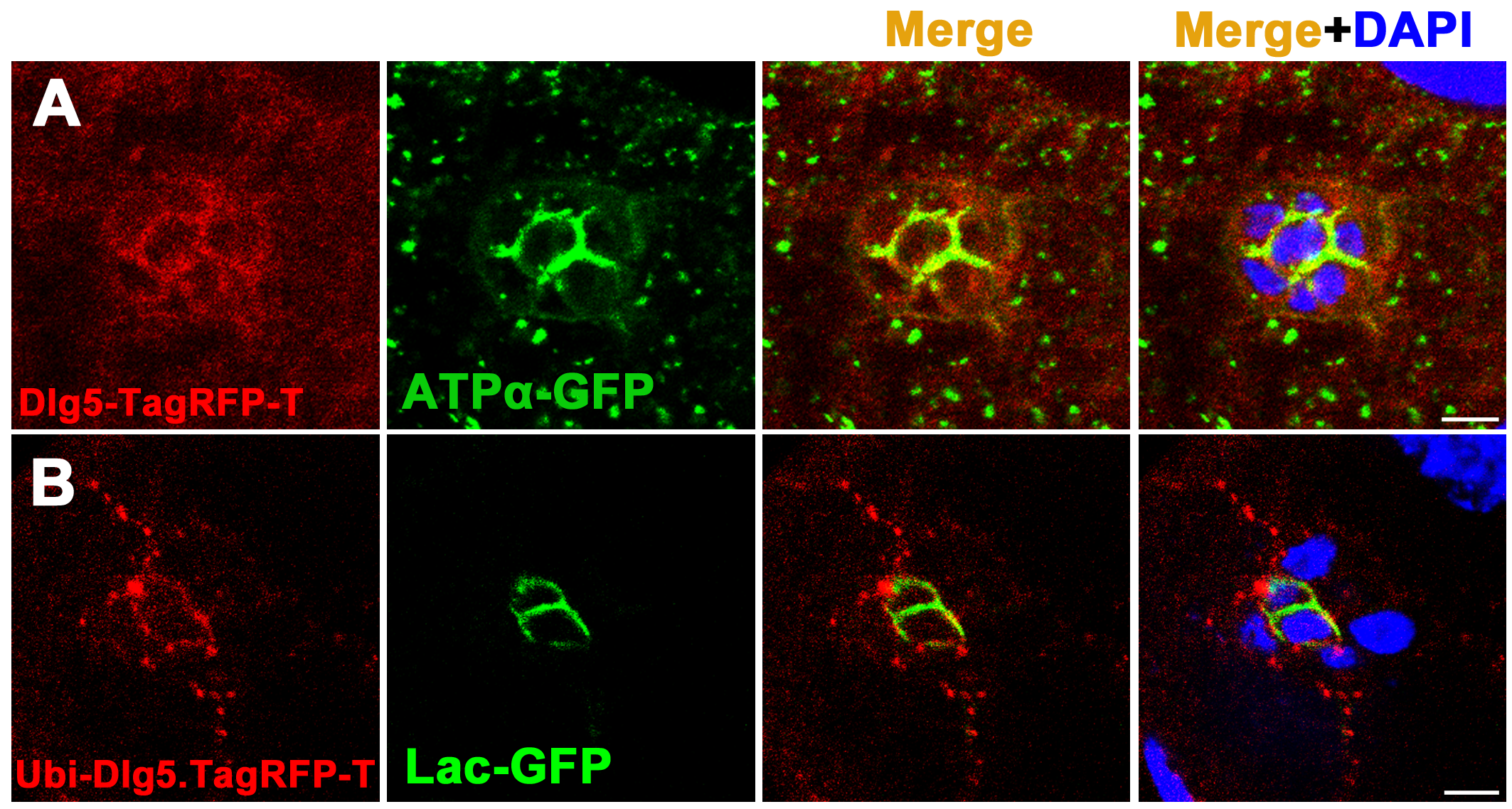

Supplement: S4 Fig — A, Dlg5-RFP (red) expressed by Dlg5-TagRFP-T colocalized with ATPα-GFP (green) in the BC cluster. B, Dlg5.TagRFP-T (red) expressed by Ubi-Dlg5.TagRFP-T partially colocalized with Lac-GFP (green). Scale bars: 10μm. (TIF) [file pone.0226061.s004.tif]

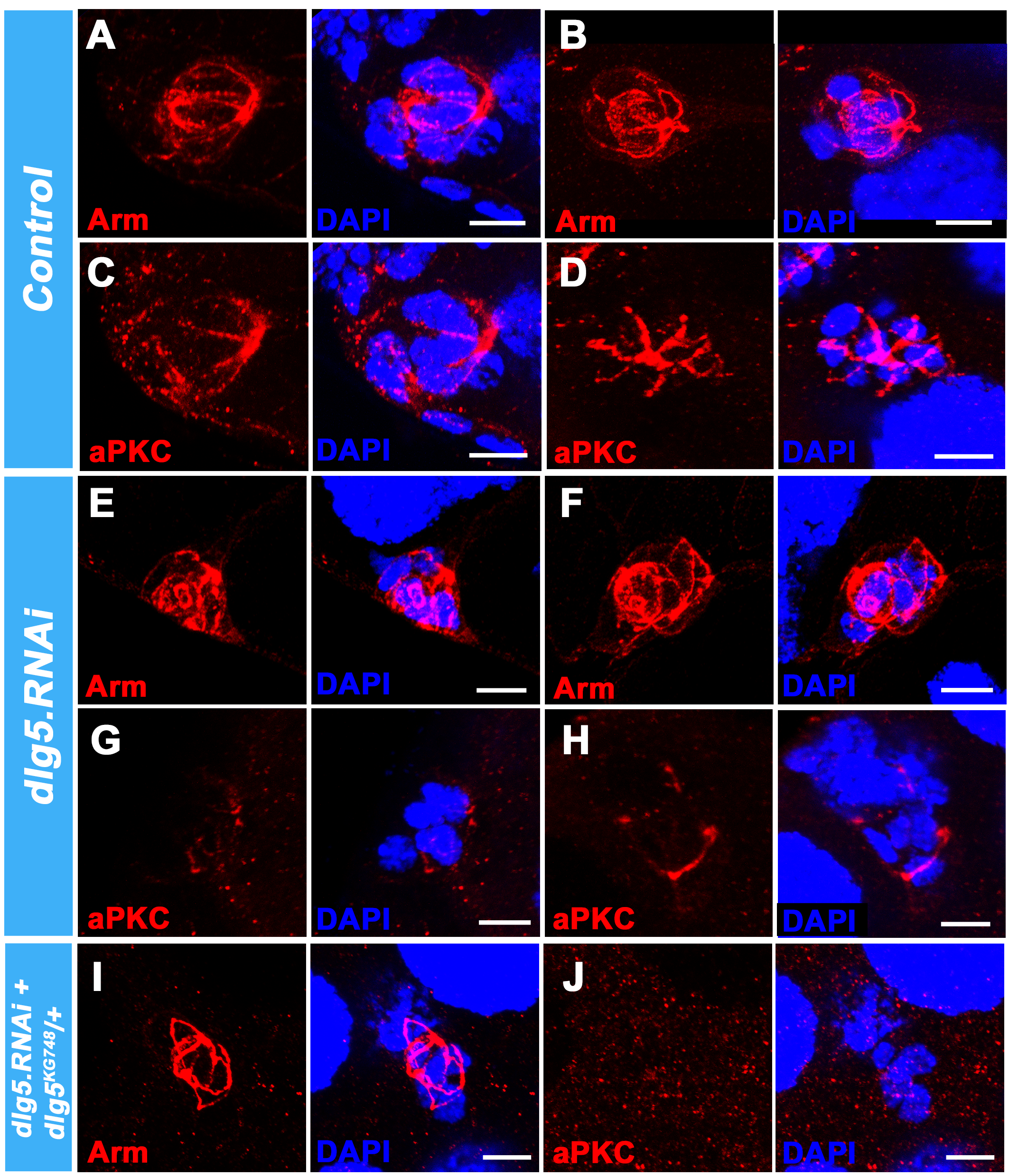

Supplement: S5 Fig — A-H, z-series projections of BC clusters corresponding to the single sections of Fig 5A–5H. A-D, In c306-Gal4/+ control BC clusters, Arm (A, B) and aPKC (C, D) displayed normal distribution in the invading stage (A, C) and the migrating stage (B, D). E-H, Expression of dlg5.RNAi driven by c306-Gal4 resulted in misdistribution of Arm (E, F) and reduction of aPKC (G, H) in both the invading stage (E, G) and the migrating stage (F, H) BC clusters compared with the c306-Gal4/+ controls (A-D). I and J, The phenotypes were more severe in dlg5 RNAi BCs combined with dlg5KG748 heterozygous background. Especially, the localization of aPKC was almost completely lost (J). DAPI was marked by blue. Arm and aPKC were marked by red, and labelled individually in the red channel. Scale bars: 10μm. (TIF) [file pone.0226061.s005.tif]

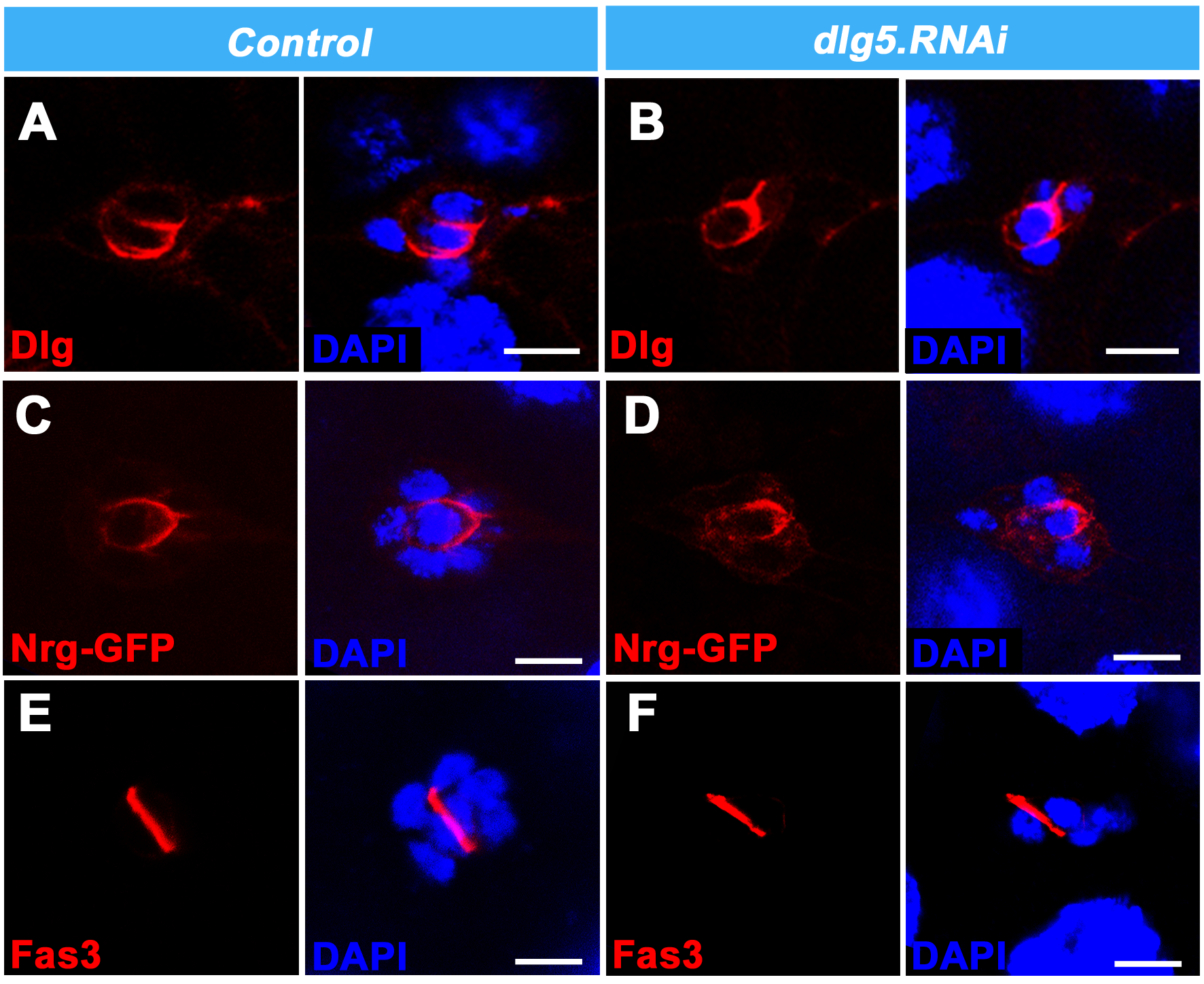

Supplement: S6 Fig — A and B, The localization of Dlg (red) was not affected in dlg5.RNAi BC cluster (B) compared to the control (A). C and D, The localization of Fas3 (red) was not affected in dlg5.RNAi BC cluster (D) compared to the control (C). E and F, The localization of Nrg-GFP (red) was not affected in dlg5.RNAi BC cluster (F) compared to the control (E). DAPI was marked by blue. Scale bars: 10μm. (TIF) [file pone.0226061.s006.tif]

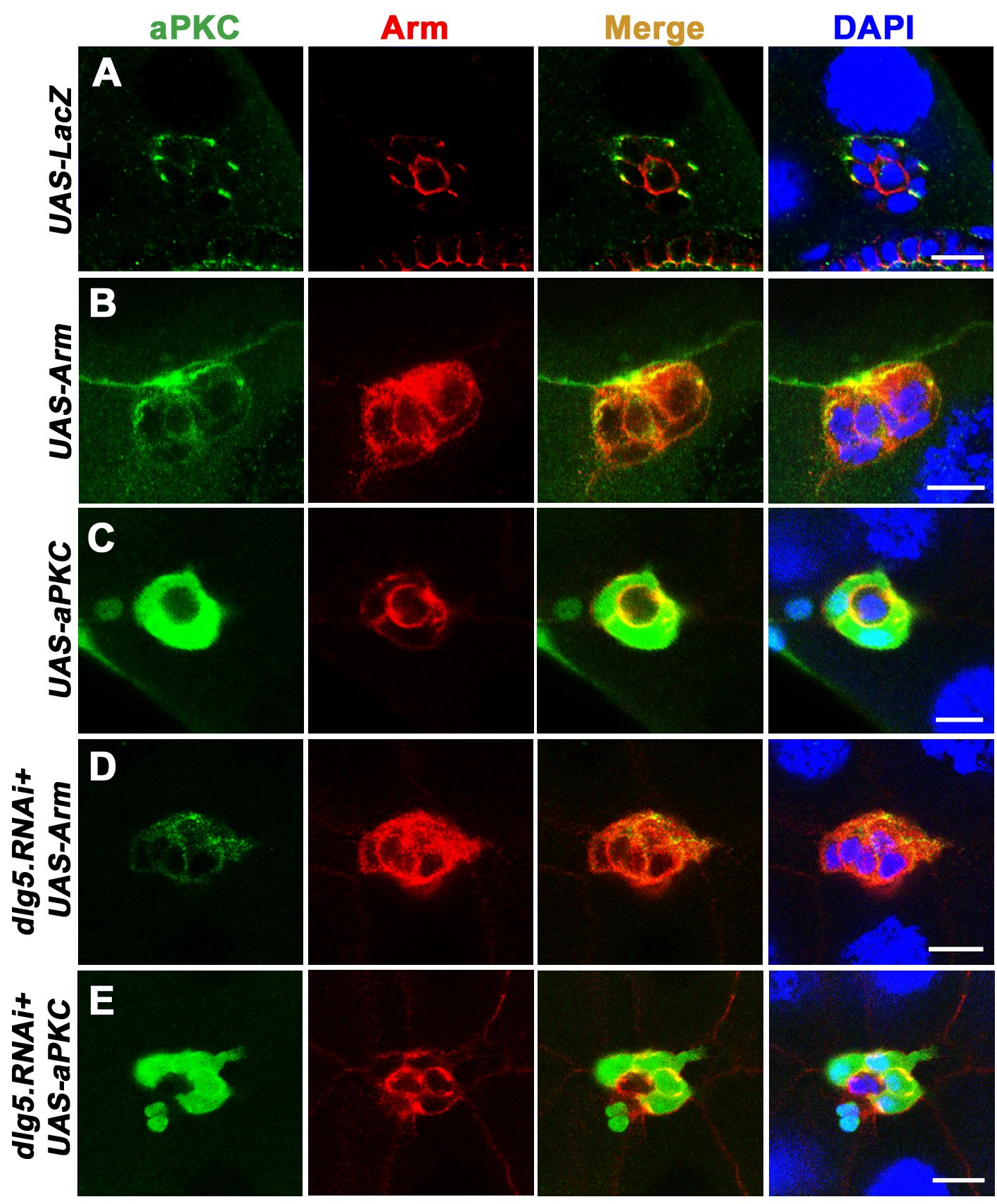

Supplement: S7 Fig — A, UAS-LacZ control shows normal localization of aPKC (green) and Arm (red). B, Overexpression of Arm causes strong enrichment of Arm in membrane and cytosol. C, Overexpression of aPKC causes strong enrichment of aPKC in the whole cell. D, The reduction of aPKC caused by dlg5.RNAi could not be rescued by overexpression of Arm. E, The misdistribution of Arm caused by dlg5.RNAi could not be rescued by overexpression of aPKC. DAPI is marked by blue. Scale bars: 10μm. (TIF) [file pone.0226061.s007.tif]

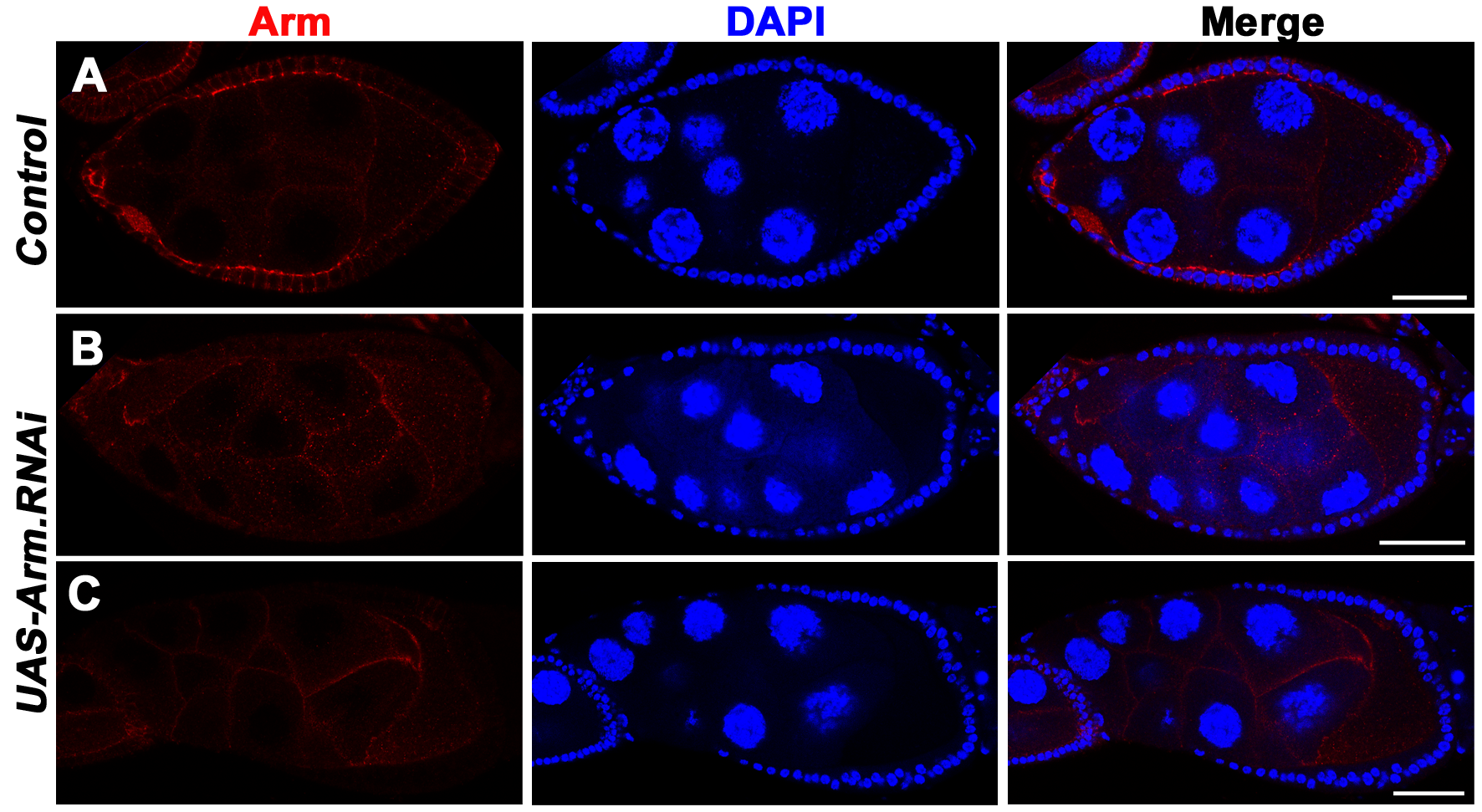

Supplement: S8 Fig — A, UAS-LacZ control shows normal expression of Arm. B and C, Efficient knockdown of Arm expression using UAS-Arm.RNAi. Scale bars: 50μm. (TIF) [file pone.0226061.s008.tif]

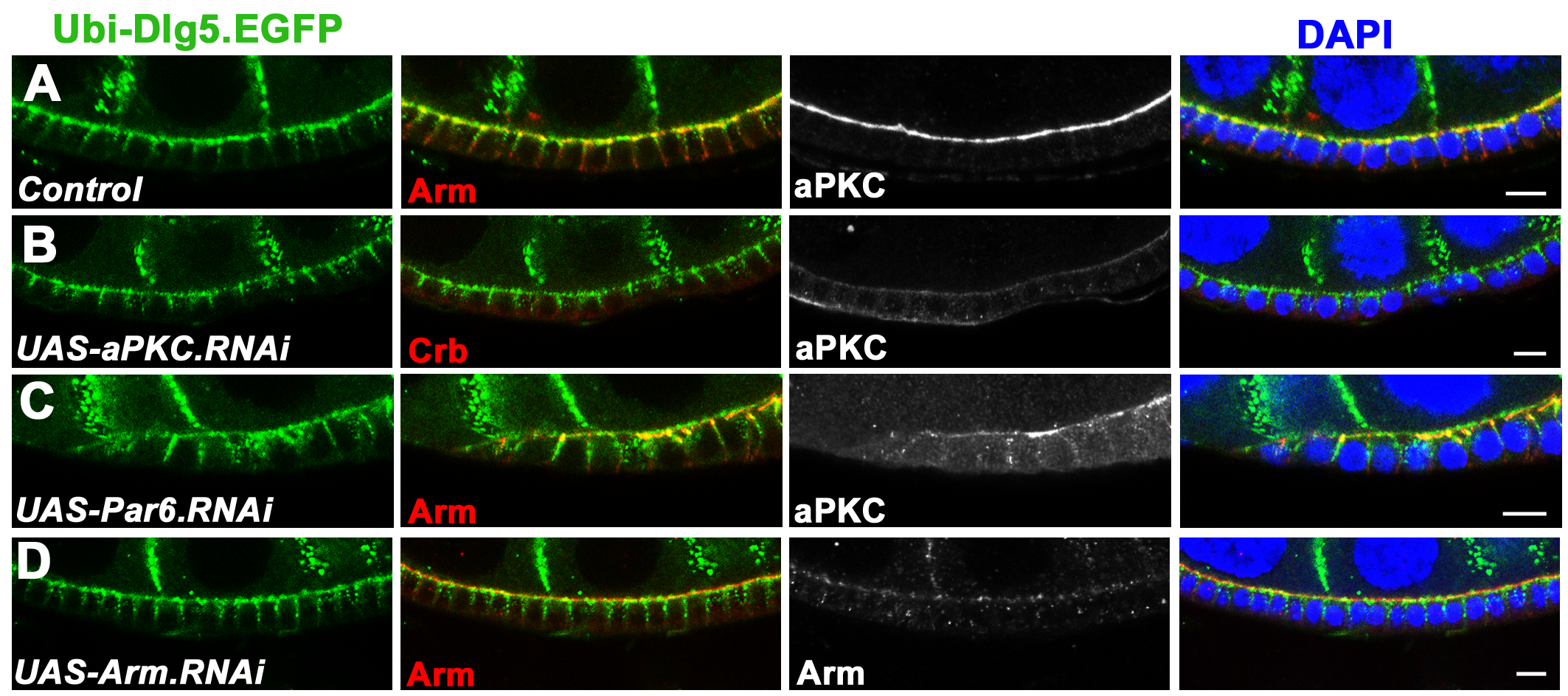

Supplement: S9 Fig — A, act5C-Gal4, tub-Gal80ts control shows apical enrichment of Dlg5.GFP expressed by Ubi-Dlg5.EGFP in follicle cells. B-D, the distribution of Dlg5.GFP is not affected by RNAi knockdown of aPKC (B), Par6 (C) or Arm (D) in follicle cells. Scale bars: 10μm. (TIF) [file pone.0226061.s009.tif]

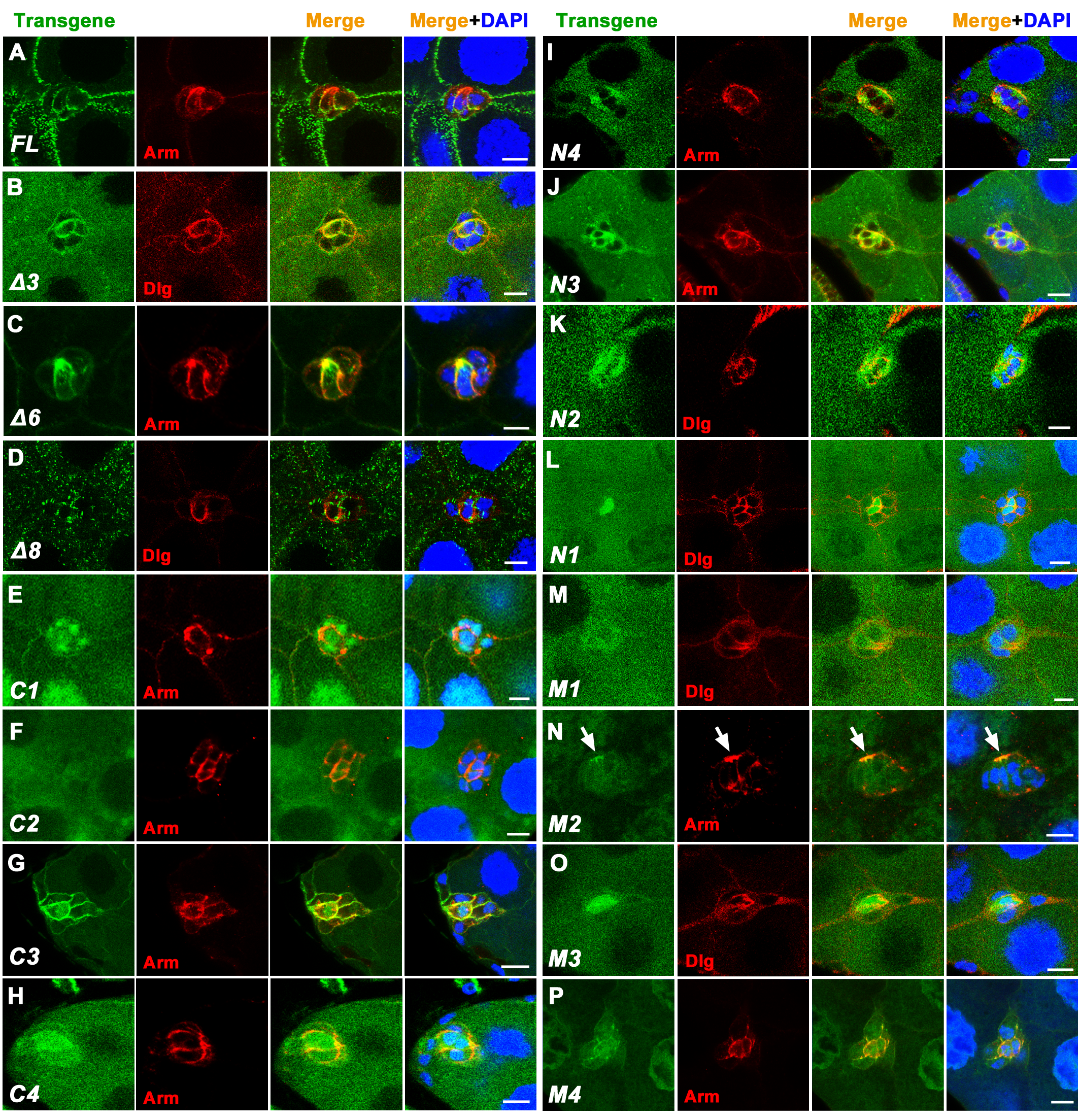

Supplement: S10 Fig — A-P, Subcellular localization of Dlg5 truncated proteins (FL, Δ3, Δ6, Δ8, C1, C2, C3, C4, N4, N3, N2, N1, M1, M2, M3, M4) in BC clusters (green). Schematics of these truncated forms were shown in Fig 8. Co-staining of polarity markers, Arm or Dlg, was shown in the second column (red). Summary of these Dlg5 truncated transgenes’ localization was shown in S3 and S4 Tables. DAPI is marked by blue. Scale bars: 10μm. (TIF) [file pone.0226061.s010.tif]
