# Supplementary material for: The polarity protein Dlg5 regulates collective cell migration during Drosophila oogenesis
Source: PLoS One. 2019 Dec 19;14(12):e0226061. doi: 10.1371/journal.pone.0226061 (PMC6922378; doi:10.1371/journal.pone.0226061)
Supplement: S1 Table — (DOCX) [file pone.0226061.s011.docx]

Table S1. Genotypes used in this study.

| Fig 1A | yw hs-Flp/+; FRT40A/Ubi-GFP FRT40A |
| --- | --- |
| Fig 1B | yw hs-Flp/+; dlg5[KG748] FRT40A/Ubi-GFP FRT40A |
| Fig 1C-E | yw hs-Flp/+; dlg5[EP2087] FRT40A/Ubi-GFP FRT40A |
| Fig 1F | c306-Gal4/UAS-Dcr2 |
| Fig 1G-I | c306-Gal4/UAS-Dcr2; UAS-dlg5.RNAi/+ |
| Fig 1J-K | c306-Gal4/UAS-Dcr2; UAS-dlg5.RNAi/+; UAS-aPKC/+ |
| Fig 1L-M | c306-Gal4/UAS-Dcr2; UAS-dlg5.RNAi/+; UAS-Arm/+ |
| Fig 2A-K | Nrg-GFP/Nrg-GFP |
| Fig 2L | Ecad-GFP/Ecad-GFP |
| Fig 3A | Dlg5-TagRFP-T/FM7; Ecad-GFP/+ |
| Fig 3B, E, F | Dlg5-TagRFP-T/Nrg-GFP |
| Fig 3C, D | Dlg5-TagRFP-T/FM7; Arm-GFP/+ |
| Fig 3G, I | Dlg5-TagRFP-T/FM7 |
| Fig 3H, J | Dlg5-TagRFP-T/FM7; Jupiter-GFP/+ |
| Fig 4A | Dlg5-TagRFP-T/FM7; Ubi-Dlg5.EGFP/+ |
| Fig 4B | Ubi-Dlg5.EGFP/Sqh-mCherry |
| Fig 4C-F’ | dlg5[EP2087] FRT40A/ dlg5[EP2087] FRT40A; Ubi-Dlg5.EGFP/+ |
| Fig 4G | dlg5[KG748] FRT40A, Ubi-Dlg5.TagRFP-T/dlg5[KG748] FRT40A, Ubi-Dlg5.TagRFP-T |
| Fig 4H-H’ | Ubi-Dlg5.TagRFP-T/GalT-GFP |
| Fig 5A-D | c306-Gal4/UAS-Dcr2 |
| Fig 5E-H | c306-Gal4/UAS-Dcr2; UAS-dlg5.RNAi/+ |
| Fig 5I-J | yw hs-Flp/+; dlg5[EP2087] FRT40A/Ubi-GFP FRT40A |
| Fig 6A | Act5C-Gal4, tub-Gal80ts/+; UAS-LacZ/Ubi-Dlg5.EGFP |
| Fig 6B | Act5C-Gal4, tub-Gal80ts/+; UAS-aPKC/Ubi-Dlg5.EGFP /+ |
| Fig 6C | Act5C-Gal4, tub-Gal80ts/+; UAS-Par6.mCherry/ Ubi-Dlg5.EGFP |
| Fig 6D | Act5C-Gal4, tub-Gal80ts/UAS-aPKC.RNAi; Ubi-Dlg5.EGFP /+ |
| Fig 6E | Act5C-Gal4, tub-Gal80ts/UAS-Par6.RNAi; Ubi-Dlg5.EGFP /+ |
| Fig 6F | Act5C-Gal4, tub-Gal80ts/UAS-Arm.RNAi ; Ubi-Dlg5.EGFP /+ |
| Fig 7B | Ubi-Dlg5.Δ1.mRuby /+ |
| Fig 7C | Ubi-Dlg5.Δ2.mRuby /+ |
| Fig 7D | Ubi-Dlg5.Δ4.EGFP /+ |
| Fig 7E | Ubi-Dlg5.Δ5.mRuby /+ |
| Fig 7F | Ubi-Dlg5.Δ7.EGFP /+ |
| Fig 8A | yw hs-Flp/+; dlg5[EP2087] FRT40A/Ubi-RFP FRT40A; Ubi-Dlg5.EGFP/+ |
| Fig 8B | yw hs-Flp/+; dlg5[EP2087] FRT40A/Ubi-RFP FRT40A; Ubi-Dlg5.EGFP/+ |
| Fig 8C | yw hs-Flp/+; dlg5[EP2087] FRT40A/Ubi-GFP FRT40A; Ubi-Dlg5.C4.mRuby/+ |
| Fig 8D | yw hs-Flp/+; dlg5[EP2087] FRT40A/Ubi-GFP FRT40A; Ubi-Dlg5.Δ1.mRuby /+ |
| Fig 8E | yw hs-Flp/+; dlg5[EP2087] FRT40A/Ubi-GFP FRT40A; Ubi-Dlg5.Δ2.mRuby /+ |
| Fig 8F | yw hs-Flp/+; dlg5[EP2087] FRT40A/Ubi-GFP FRT40A; Ubi-Dlg5.Δ5.mRuby /+ |
| Fig 8G | yw hs-Flp/+; dlg5[EP2087] FRT40A/Ubi-GFP FRT40A; Ubi-Dlg5.Δ6.mRuby /+ |
| Fig 8H | yw hs-Flp/+; dlg5[EP2087] FRT40A/Ubi-RFP FRT40A; Ubi-Dlg5.Δ7.EGFP /+ |
| Fig 8I | yw hs-Flp/+; dlg5[EP2087] FRT40A/Ubi-RFP FRT40A; Ubi-Dlg5.Δ8.EGFP /+ |
| Fig 8J,J’ | Ecad-GFP/Ecad-GFP |
| Fig 8K,K’ | dlg5[KG748] FRT40A Ecad-GFP/ dlg5[KG748] FRT40A Ecad-GFP; Ubi-Dlg5.C4.mRuby/+ |
| S1B Fig | yw hs-Flp tub-Gal4 UAS-GFP/yw ey-Flp; tub-Gal80 FRT40A/FRT40A |
| S1C Fig | yw hs-Flp tub-Gal4 UAS-GFP/ yw ey-Flp; tub-Gal80 FRT40A/taiman[k15101] FRT40A |
| S1D Fig | yw hs-Flp tub-Gal4 UAS-GFP/ yw ey-Flp; tub-Gal80 FRT40A/Scim13[1] FRT40A |
| S1E Fig | yw hs-Flp tub-Gal4 UAS-GFP/ yw ey-Flp; tub-Gal80 FRT40A/Rack1[EY128] FRT40A |
| S1F Fig | yw hs-Flp tub-Gal4 UAS-GFP/ yw ey-Flp; tub-Gal80 FRT40A/dlg5[KG748] FRT40A |
| S1G-G’’ Fig | CG31689[CB03239]/+ |
| S1H-H’’ Fig | CG31689[CB03239]/+ |
| S2A Fig | UAS-Dcr2/+; slbo-Gal4 UAS-GFP/+ |
| S2B-C’ Fig | UAS-Dcr2/+; slbo-Gal4 UAS-GFP/UAS-dlg5.RNAi |
| S2D Fig | UAS-Dcr2/upd-Gal4; slbo-Gal4 UAS-GFP/+ |
| S2E-F’ Fig | UAS-Dcr2/upd-Gal4; slbo-Gal4 UAS-GFP/UAS-dlg5.RNAi |
| S3A-C Fig | w1118 |
| S4A Fig | Ubi-Dlg5.TagRFP-T/+; ATPalpha-GFP/+ |
| S4B Fig | Ubi-Dlg5.TagRFP-T/Lac-GFP |
| S5A-D Fig | c306-Gal4/UAS-Dcr2 |
| S5E-H Fig | c306-Gal4/UAS-Dcr2; UAS-dlg5.RNAi/+ |
| S5I J Fig | c306-Gal4/UAS-Dcr2; UAS-dlg5.RNAi/dlg5[KG748] FRT40A |
| S6A C E Fig | c306-Gal4/UAS-Dcr2 |
| S6B D F Fig | c306-Gal4/UAS-Dcr2; UAS-dlg5.RNAi/+ |
| S7A Fig | Act5C-Gal4, tub-Gal80ts/+; UAS-LacZ/+ |
| S7B Fig | Act5C-Gal4, tub-Gal80ts/+; UAS-Arm/+ |
| S7C Fig | Act5C-Gal4, tub-Gal80ts/+; UAS-aPKC/+ |
| S7D Fig | Act5C-Gal4, tub-Gal80ts/UAS-dlg5.RNAi; UAS-Arm/+ |
| S7E Fig | Act5C-Gal4, tub-Gal80ts/UAS-dlg5.RNAi; UAS-aPKC/+ |
| S8A Fig | Act5C-Gal4, tub-Gal80ts/UAS-LacZ |
| S8B C Fig | Act5C-Gal4, tub-Gal80ts/UAS-Arm.RNAi |
| S9A Fig | Act5C-Gal4, tub-Gal80ts/+ |
| S9B Fig | Act5C-Gal4, tub-Gal80ts/UAS-aPKC.RNAi |
| S9C Fig | Act5C-Gal4, tub-Gal80ts/UAS-Par6.RNAi |
| S9D Fig | Act5C-Gal4, tub-Gal80ts/UAS-Arm.RNAi |
| S10A Fig | Ubi-Dlg5.EGFP/+ |
| S10B Fig | Ubi-Dlg5.Δ3.mRuby/+ |
| S10C Fig | Ubi-Dlg5.Δ6.mRuby/+ |
| S10D Fig | Ubi-Dlg5.Δ8.EGFP/+ |
| S10E Fig | Ubi-Dlg5.C1.EGFP/+ |
| S10F Fig | Ubi-Dlg5.C2.mRuby/+ |
| S10G Fig | Ubi-Dlg5.C3.EGFP/+ |
| S10H Fig | Ubi-Dlg5.C4.mRuby/+ |
| S10I Fig | Ubi-Dlg5.N4.EGFP /+ |
| S10J Fig | Ubi-Dlg5.N3.EGFP/+ |
| S10K Fig | Ubi-Dlg5.N2.EGFP/+ |
| S10L Fig | Ubi-Dlg5.N1.EGFP/+ |
| S10M Fig | Ubi-Dlg5.M1.EGFP/+ |
| S10N Fig | Ubi-Dlg5.M2.EGFP/+ |
| S10O Fig | Ubi-Dlg5.M3.EGFP/+ |
| S10P Fig | Ubi-Dlg5.M4.EGFP/+ |
