# Supplementary material for: The polarity protein Dlg5 regulates collective cell migration during Drosophila oogenesis
Source: PLoS One. 2019 Dec 19;14(12):e0226061. doi: 10.1371/journal.pone.0226061 (PMC6922378; doi:10.1371/journal.pone.0226061)
Supplement: S3 Table — (DOCX) [file pone.0226061.s013.docx]

Table S3. Summary of the Dlg5 deletions analyses.

| Trans-genes | Deleted domain(s) or region(s) | Localization | | | Rescue BC migration delay |
| --- | --- | --- | --- | --- | --- |
|  |  | PC-BC surfaces | PC apical domain | Punctate aggregation |  |
| FL | None | ++ | - | + | ++ |
| Δ1 | CC-PDZ1-PDZ2 | + | + | - | - |
| Δ2 | PDZ1-PDZ2 | + | +- | ++ | + |
| Δ3 | The linker region | + | ND | ± | ND |
| Δ4 | PDZ3-PDZ4-SH3-GUK | - | - | ++ | - |
| Δ5 | PDZ3-PDZ4 | + | +- | ++ | - |
| Δ6 | SH3-GUK | ++ | + | - | - |
| Δ7 | SH3 | - | - | ++ | - |
| Δ8 | GUK | - | - | ++ | - |

BC: border cells; PC: polar cells.
