# Supplementary material for: The polarity protein Dlg5 regulates collective cell migration during Drosophila oogenesis
Source: PLoS One. 2019 Dec 19;14(12):e0226061. doi: 10.1371/journal.pone.0226061 (PMC6922378; doi:10.1371/journal.pone.0226061)
Supplement: S4 Table — (DOCX) [file pone.0226061.s014.docx]

Table S4. Summary of the Dlg5 domains analyses.

| Trans-genes | Expressed domain(s) or region(s) | Localization | | | PC enrichment |
| --- | --- | --- | --- | --- | --- |
|  |  | membrane | cytosol | nucleus |  |
| C1 | GUK | - | ± | ++ | - |
| C2 | SH3-GUK | - | + | ± | - |
| C3 | PDZ4-SH3-GUK | ++ | ± | - | + |
| C4 | PDZ3-PDZ4-SH3-GUK | ± | + | ± | + |
| N4 | CC-PDZ1-PDZ2 | - | + | - | ± |
| N3 | CC | + | + | - | + |
| N2 | N-terminal 217aa | - | + | ± | - |
| N1 | N-terminal 127aa | - | + | ± | + |
| M1 | PDZ1-PDZ2 | - | + | - | - |
| M2 | The linker region | ± | + | +- | - |
| M3 | PDZ3-PDZ4 | - | + | - | + |
| M4 | The liner-PDZ3-PDZ4 | +- | + | - | - |

CC: Coiled-coil. C3 is the MAGUK Core domains.
